# Accumulation of As, Ag, Cd, Cu, Pb, and Zn by Native Plants Growing in Soils Contaminated by Mining Environmental Liabilities in the Peruvian Andes

**DOI:** 10.3390/plants10020241

**Published:** 2021-01-27

**Authors:** Edith Cruzado-Tafur, Katarzyna Bierla, Lisard Torró, Joanna Szpunar

**Affiliations:** 1 Université de Pau et des Pays de l’Adour, E2S UPPA, CNRS, IPREM UMR 5254, Hélioparc, 64053 Pau, France; em.cruzado-tafur@univ-pau.fr (E.C.-T.); katarzyna.bierla@univ-pau.fr (K.B.); 2 Geological Engineering Program, Faculty of Sciences and Engineering, Pontifical Catholic University of Peru (PUCP), Av. Universitaria 1801, San Miguel, Lima 15088, Peru; ltorro@pucp.edu.pe

**Keywords:** mining environmental liabilities, native plants, metals, translocation factor, bioconcentration factor, phytoremediation

## Abstract

The capability of native plant species grown in polluted post-mining soils to accumulate metals was evaluated in view of their possible suitability for phytoremediation. The study areas included two environmental liabilities in the Cajamarca region in the Peruvian Andes. The content of As, Ag, Cd, Cu, Pb, and Zn was determined in individual plant organs and correlated with soil characteristics. The degree of the pollution depended on the metal with results ranging from uncontaminated (Cd) to moderately (Zn), strongly (As, Cu), and extremely contaminated (Pb, Ag) soils. The metals were mainly present in the fractions with limited metal mobility. The bioaccumulation of the metals in plants as well the translocation into overground organs was determined. Out of the 21 plants evaluated, *Pernettya prostrata* and *Gaultheria glomerate* were suitable for Zn, and *Gaultheria glomerata* and *Festuca* sp. for Cd, phytostabilization. The native species applicable for Cd phytoremediation were *Ageratina glechonophylla*, *Bejaria* sp., whereas *Pernettya prostrata Achyrocline alata,* *Ageratina fastigiate*, *Baccharis alnifolia*, *Calceolaria tetragona*, *Arenaria digyna*, *Hypericum laricifolium*, *Brachyotum radula*, and *Nicotiana thyrsiflora* were suitable for both Cd and Zn. None of the studied plants appeared to be suitable for phytoremediation of Pb, Cu, As and Ag.

## 1. Introduction

Mining has been around since ancient times and has been essential for economic and industrial development of societies [1]. The extraction of natural resources has generated an economic surge in Latin America [2]. During the recent decades, the mining sector has been one of the main economic pillars of Peru [3], making it the main producer of gold, zinc, lead, and tin in Latin America and the second largest producer of copper, silver, and zinc worldwide [4].

Nevertheless, mining operations are also responsible for significant environmental damage by producing a large amount and diversity of residues, affecting the quality of water, soil, and air [5], of which soil pollution is one of the most crucial environmental problems [6]. Likewise, the abandoned mines without effective shut-down procedures are causing a nuisance in the mining districts due to metal transfer to the environment [7], being one principal reason for the emergence of conflicts in Peru [8]. Metals are persistent in the environment and, being nonbiodegradable [9], cause serious harm bioaccumulating in flora and fauna [6,10]. The installations, effluents, emissions, remains, or deposits of residues from abandoned or inactive mines constituting a permanent and potential risk for health, the surrounding ecosystem, and property are defined as mining environmental liabilities (MELs) [11,12]. The lack of adequate legislation has caused MELs to increase in number and magnitude resulting in significant environmental damage [5], generating problems to local farmers and peasants [13] and provoking resistance and opposition from local communities [2]. In 2019 in the Peruvian territory, approximately 8448 MELs [14] were identified with the highest number of them located in Ancash, Apurímac, Arequipa, Cajamarca, Huancavelica, Junín, Pasco, and Puno regions [14,15]. Although Peru has embarked on a process of regulation and institutional changes aimed at achieving adequate protection of the environment [8], these efforts are insufficient, thus increasing the demand for innovative and sustainable solutions [16]. 

Phytoremediation is considered to have many advantages over other methods, such as low cost, in situ remediation, respect for the environment, and landscape improvements [17]. Phytoremediation, including phytostabilization and phytoextraction, has shown satisfactory results and is a promising alternative relying on the natural ability of plants to recover metals from soil [9,17,18,19,20]. The uptake, translocation, and phytoaccumulation of metals from the soil depends specifically on plant species [18]. Native plant species are preferred because they are adapted to the local climatic conditions and, by growing in contaminated places, they have already proven their resistance to an excess of metals [1,21].

The study areas included two environmental liabilities in the Hualgayoc district, Cajamarca region in the Peruvian Andes. The principal problems of metal contamination in the watersheds in the Cajamarca region derive from the mining operations located at high altitudes so that in the rainy season, the mining waste goes directly to the lower agricultural valleys [22]. Within the region of Cajamarca, Hualgayoc is a complex mining district that since Spanish colonial times has been known for Ag-rich polymetallic mineralization; in addition to Zn, Pb, and Cu [23,24]. Currently, this region is one of the most affected by the presence of mining environmental liabilities [25]. The Hualgayoc district has a mostly rural population dedicated to agriculture; the mining sector represents a great possibility of economic development for this area, however, fears persist regarding an increase in environmental pollution [25]. In this context, the main objective of this research was to evaluate the capacity of bioaccumulation of As, Ag, Cd, Cu, Pb, and Zn in tissues of native plants as well the translocation into over-ground organs from polluted post-mining soils. The purpose was to establish the potential of different plants species for phytoremediation in post-mining sites.

## 2. Materials and Methods

### 2.1. Study Area and Sampling

The sampling sites were located in the Andes of northern Peru, in the Hualgayoc district, in the west of the province of Hualgayoc, between the districts of Chugur and Bambamarca in the department of Cajamarca.

Hualgayoc district is located in the west of the province of Hualgayoc in the department of Cajamarca in the Andes of Northern Peru. It is ca. 45 km away, as the bird flies, from Cajamarca city and 865 km by road from Lima city [25]. Since Spanish colonial times, Hualgayoc has been famous for Ag-rich polymetallic mineralization, in addition to Zn, Pb, and Cu [23,24,26]. The main mineralized structures in the Hualgayoc district are mantos and veins hosted largely in the upper Goyllarisquizga Group, the Inca Formation and lower Chulec Formation rocks [23,24,26]. The Hualgayoc district has 943 MELs [14], two of them belonging to the ex-mining unit Los Negros have been studied here; they are found on the left bank and downstream from the Hualgayoc river creek in the flak of the Llaucano river valley. The identity of the liable parties of the generation of these MELs have not been identified yet [14]. In order to evaluate the environmental impact of the abandoned mines the two chosen sampling sites included a leach pad (S1) located on top of a mine waste deposit and a mine dump (S2) made up of clay gravel with sand.

**Figure 1:**
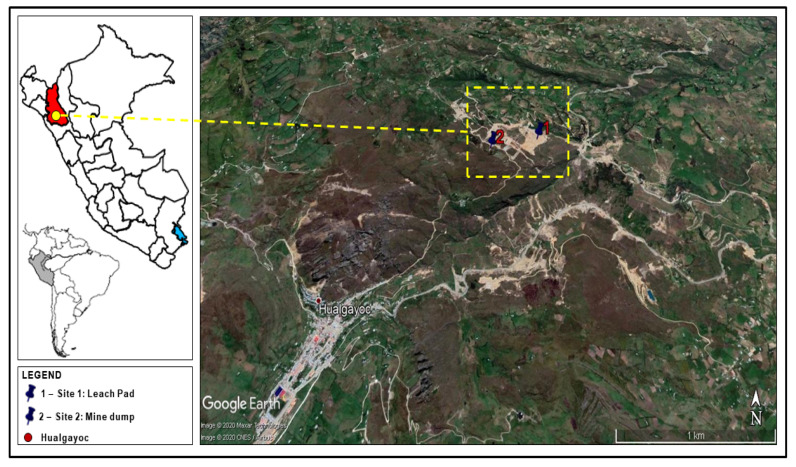
Localization of the mining environmental liabilities (site #1—leach pad, site #2—mine dump) of the ex-mining unit Los Negros in Hualgayoc district, Cajamarca region, Peru.

Sampling area #1 leach pad (a latitude 6°44′49″ S, longitude 78°35′36″ W, and altitude of 3249 m above sea level) was located on top of a mine waste deposit and had no drainage. Sampling area #2 mine dump (latitude 6°44′53″ S, longitude 78°35′49″, and altitude of 3399 m above sea level) had granular material made up of clay gravel with sand, no stabilization work was observed and there was no revegetation nor drainage (Figure 1).

Soil and plant samples were taken following the criteria based on the distance from the point of contamination, wind direction, slope and inclination, vegetation cover, and soil texture [27]. The location of the points for soil sampling in both sites was fixed at 0, 15, 30, 45 and 60 m away from the point of contamination, forwards downhill slide. For the determination of soil profile characteristics, samples were taken at two depths between 0–15 and 15–30 cm intervals. Following this pattern, a total of 20 soil samples were collected from the two sites. Plants and soil samples were collected in July 2018 (dry season). Sampling of flora specimens was carried out in the same locations as for soil sampling. To do so, an area of 4 m^2^ around each soil sampling point was delimited. Each plant was stored in polyethylene bags before being carried to the laboratory. A total of 47 plant samples (from 1 to 5 replicates) were collected from the two sampling sites, including 29 samples from site #1, and 18 samples from #2. Five species were found in both areas so a total of 21 unique species of native flora have been identified. The taxonomic identification of the collected plants was carried out by Dr. Manuel Timaná (the Pontifical Catholic University of Peru, Peru) and Mg. Paul Gonzales Arce (the Laboratory of Floristics of the Herbarium of the Natural History Museum of the Universidad Nacional Mayor de San Marcos, Peru).

### 2.2. Chemical Analysis of Soils

The soil samples were analyzed for pH, electrical conductivity, organic matter and texture at the Soil, Plant, Water and Fertilizer Analysis Laboratory of the Universidad Nacional Agraria La Molina in Lima (Peru). pH was measured using a suspension of soil in deionized water at a ratio 1:1 using a Consort pH-meter (Turnhout, Belgium) and electrical conductivity was measured in aqueous extract of soil in water at a ratio 1:1. Organic matter content was determined using the Walkley and Black method [28] based on the oxidation of organic carbon with potassium dichromate, sulfuric acid, and concentrated phosphoric acid. Texture evaluation was conducted using the classical Bouyoucos Hydrometer method—the mechanical analysis of soils utilising the suspension of solids for the quantification of the content of sand, silt, and clay in percentage [29].

Metal content in soils was analyzed at the SGS Geochemical Assays Laboratory, Lima, Peru. Approximately 0.20 g of soil with particle size below 106 µm was digested in two steps in Hot Blocks (Environmental Express, Charleston, SC, USA). For the first digestion step (at 90 °C for 30 min), 1 mL of concentrated nitric acid was added; for the second one (at 90 °C for 1 h), 3 mL of concentrated hydrochloric acid was added. Once the digest cooled to room temperature, 2 mL of concentrated hydrochloric acid was added and the mixture was diluted with ultrapure water (18.2 MΩ·cm) up to 20 mL. The concentration of metals was determined by an inductively coupled plasma emission spectrometer (ICP-OES) using a Perkin Elmer Model Optima 8300 DV instrument. The certified soil reference materials OREAS 906, OREAS 907, and OREAS 522 (from ORE Research & Exploration Pty Ltd., Bayswater North, Victoria, Australia) were used for quality control.

### 2.3. Calculation of the Geo-Accumulation Index (I_geo_)

The geo-accumulation index (*I_geo_*) was introduced by Muller [30] for the evaluation accumulation of metals in river sediments. Since then it has been adopted by many researchers in order to classify sediments and soils from the point of view of metal pollution level. The values necessary to calculate *I_geo_* are concentration of metal in given soil and the background level concentrations. In our study, baseline values obtained by Santos-Francés et al. [22] from La Zanja region at ca. 30 km from the sampling place are used for calculation purposes, except for silver, for which the natural abundance in the Earth’s crust was used [31]. The values used for calculations are given in Supplementary Information (Table S1).

The *I_geo_* was calculated according to the equation:

(1) $$I_{geo}=log_{2}\;\frac{C_{i}}{1.5*B_{i}}$$

where *C_i_* is the measured concentration of the *i* metal examined in the soil and *B_i_* is the background level of this metal. The factor 1.5 is used to correct possible variations in the background values of a particular metal in the environment.

Depending on the value of the *I_geo_*, the soils are classified into seven groups [32]: unpolluted (*I_geo_* < 0), unpolluted to moderately polluted (0 ≤ *I_geo_* < 1), moderately polluted (1 ≤ *I_geo_* < 2), moderately to strongly polluted (2 ≤ *I_geo_* < 3), strongly polluted (3 ≤ *I_geo_* < 4), strongly to very strongly polluted (4 ≤ *I_geo_* < 5), and very strongly polluted (*I_geo_* ≥ 5).

### 2.4. Sequential Extraction Procedure

In order to identify how the metals are distributed among the soil components, the soil samples were subjected to sequential extraction based on the Tessier approach [33], using the modified method [34] adapted to the soil parameters consisting of the four following steps:

1. Exchangeable (loosely bound) metals fraction: 15 mL 1 M CH_3_COONH_4_, pH 7 (shaking time 1 h at room temperature) followed by the addition of 30 mL 1 M CH_3_COONa by acidification with CH_3_COOH to pH 5 (shaking time 5 h at room temperature).
2. Metal bound with hydrated iron and manganese oxides: 30 mL 0.04 M NH_2_OH·HCl in 25% (*v*/*v*) CH_3_COOH (shaking time 5 h at 95 °C).
3. Metals bound with organic matter: 7.5 mL 0.02 M HNO_3_ + 7.5 mL 30% H_2_O, pH 2 (shaking time 2 h at 85 °C), then 7.5 mL 30% H_2_O_2_, pH 2 was added (shaking time 3 h at 85 °C) and finally, 15 mL 3.2 M CH_3_COONH_4_ in 20% (*v*/*v*) HNO_3_ was added (shaking time 0.5 h at room temperature).
4. Metals bound to mineral fraction: 4.5 mL 10M HNO_3_ + 3 × 3 mL H_2_O_2_ time 1 h, then 15 mL H_2_O (shaking time 0.5 h at 95 °C) was added.

All samples were centrifuged at 400 rpm for 10 min and filtered (0.45 µm). Metals present in the supernatants were quantified by ICP mass spectrometry (ICP-MS) using the reaction cell mode pressurized with He and H_2_ gas. The dilutions performed allowed work in the calibration range of 0.01–10 ppb; an 8-point calibration curve was used. The isotopes monitored were ^107^Ag, ^109^Ag; ^75^As; ^111^Cd, ^112^Cd, ^114^Cd; ^63^Cu, ^65^Cu; ^206^Pb, ^207^Pb, ^208^Pb; ^64^Zn, ^66^Zn, and ^68^Zn. Analytical blanks were run in parallel.

### 2.5. Chemical Analysis of Plants

The vegetal material was washed with tap water (for the disposal of soil remains) and then with distilled water. All the plant samples were separated into leaves, stems, and roots, which were dried at 30–40 °C. Foliar samples were crushed and ground, and 0.10 g subsamples were digested in the heat block (SCP Science, Courtaboeuf, France) in triplicate. The digestion was carried out in two steps: the first one using 2 mL of nitric acid (70%) at 85 °C for 4 h 30 min and the second one using 0.75 mL of hydrogen peroxide (30%) at 85 °C for 4 h 30 min. The digest was diluted to 50 mL with ultrapure water (18.2 MΩ·cm). The elemental composition was analyzed by ICP mass spectrometry (ICP-MS) using an Agilent 7500 (Agilent, Tokyo, Japan) instrument equipped with a reaction cell pressurized with He and H_2_ gas. The dilutions performed allowed work in the calibration range of 0.1–10 ppb. A 7-point calibration curve was used. The isotopes monitored were ^107^Ag, ^109^Ag; ^75^As; ^111^Cd, ^112^Cd, ^114^Cd; ^63^Cu, ^65^Cu; ^206^Pb, ^207^Pb, ^208^Pb; ^64^Zn, ^66^Zn, and ^68^Zn. Analytical blanks were analyzed in parallel. The Standard Reference Material 1573a Tomato Leaves of the NIST (Gaithersburg, MD 20899, USA) was used for quality control.

### 2.6. Calculation of the Translocation Factor (TF) and the Bioconcentration Factor (BCF)

The translocation factor (TF) and the bioconcentration factor (BCF) were calculated to assess the phytoremediation potential of the studied plants. The BCF is defined as the ratio of the metal content accumulated in the plant to the content in the soil [35], as given below:

BCF= C_p_/C_so_ (2)

where C_p_ is the metal concentration in the plant (shoot) and C_so_ is the total metal concentration in the soil. Plants with a BCF value higher than one are considered to be suitable candidates for phytoextraction [36,37].

The ability of the trace metal transfer from the roots to the aerial part is expressed using the translocation factor TF [38], calculated as follows:

TF = C_s_/C_r_ (3)

where C_s_ is the metal concentration the aerial part (shoot) of the plant and C_r_ is the concentration in roots. Plants with TF values higher than one can be potentially used for phytoextraction [38].

### 2.7. Statistics

The measurements were carried out in triplicates and the results with relative standard deviation higher than 10% were discarded and the measurements repeated. Values are reported as mean ± standard deviation (SD) of three replications.

In order to assess the differences in *I_geo_* values for the studied elements between the soil samples at each site, we first performed a normal test to determine whether the data was normally distributed. We found that the null hypothesis of normal distribution could not be rejected for any of the samples. Therefore, we decided to apply a *t*-test in order test for differences between each pair of elements. We applied the Bonferroni correction for multiple testing, which resulted in a significance threshold of alpha = 0.01/n, where n = 15 was the number of possible pairs of elements.

## 3. Results and Discussion

### 3.1. Soil Physicochemical Characterization

Physicochemical properties of the soils are given in Table 1**.** The two sites had similar extremely acidic pH values in the range of 3.59–4.19 in site #1 and 2.77–4.02 in site #2. As a result, no carbonates and low organic matter contents were present. Soil was non-saline and had low electrical conductivity (EC) with an average value of 0.08 dSm^−1^ in site #1 and of 0.15 dSm^−1^ in site #2. In the texture of soils, the sand fraction dominated at 42–66% in site #1 and at 52–62% in site #2, varying from sandy clay loam to clay loam textures in the different layers. The mineralogical composition of soils was dominated by illite/sericite, kaolinite, quartz, and jarosite [39]. The most important factor affecting trace metals availability is soil pH [1], which usually increases the mobility and bioavailability of the metal with lower pH values, with the content of organic matter and clay minerals [10,40]. On the other hand, soils rich in organic matter can bind metals efficiently in acidic conditions, however, bonding capacity depends on the metal [10]. The samples had fine granulometry due to the major presence of sandy fraction; the fine soil particles can be easily transported by wind and water erosion to the nearby environment [41]. The acidity of the soil and their unbalanced properties pointed to the poor soil management [42].

Santos-Francés et al. [22] at Colquirrumi mine, located 6 km NW of our study area, reported acid soils (pH = 4.6), low organic matter content (5.04%), and silt sand texture (29.5% sand, 41.5 silt, and 30.8% clay); also, soils Colquirrumi are on quartzite and dacite substrates, which are much less reactive [39]. These results show significant differences in comparison with the study reported by Bech et al. [43] for the Carolina Mine, located 4 km SW of the study area, which reported basic pH of soil (i.e., pH = 6.8–8.0), high calcium carbonates levels, slight electrical conductivity (0.1–0.8 dSm^−1^), and silt loam texture.

### 3.2. Soil Metal Composition

**Figure 2:**
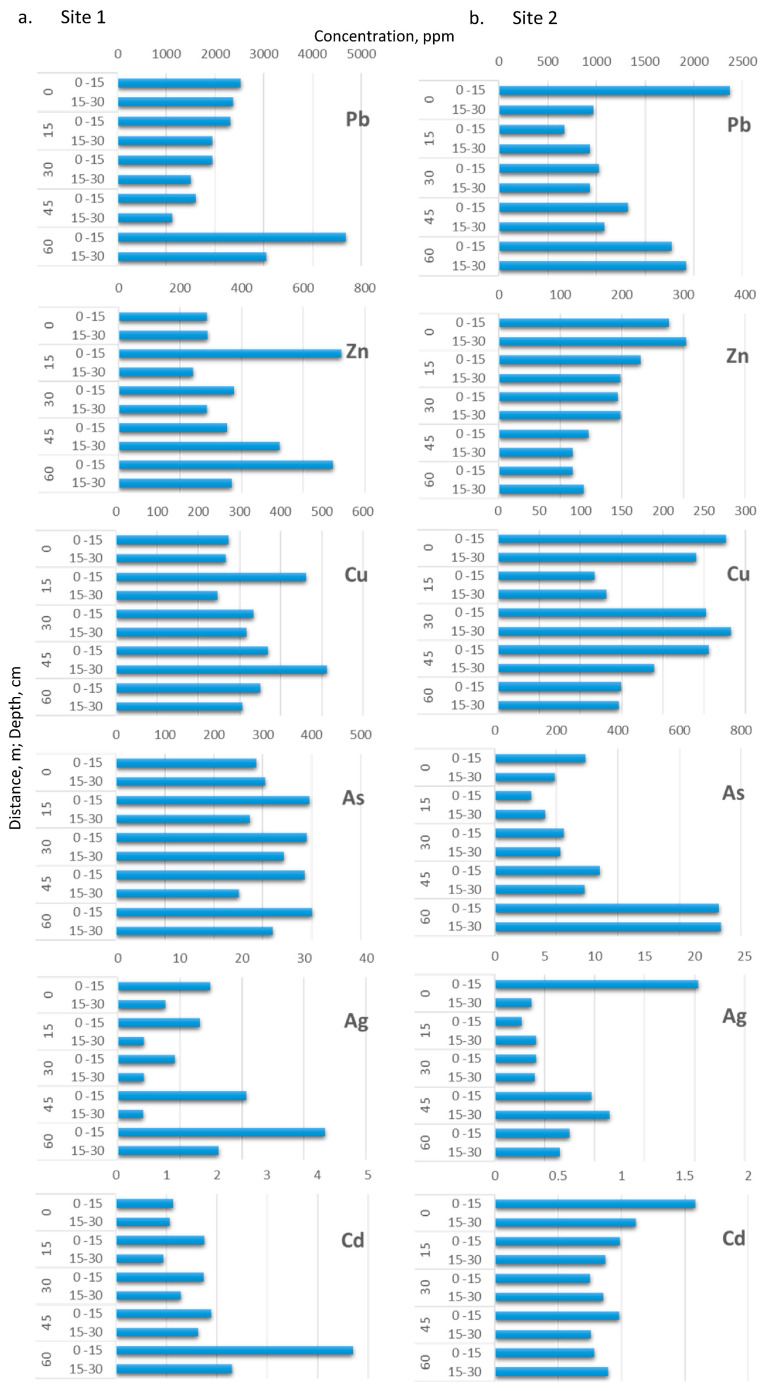
Soil Pb, Zn, As, Cu, Ag, and Cd concentration (mg kg^−1^) for five distances (0–60 m) ant two depths (0–15 and 15–30 cm) in site#1 and site #2 from Hualgayoc district, Cajamarca region, Peru.

Following the initial screening of 34 elements [39], the study of Pb, Zn, As, Cu, Ag, and Cd was found of the highest interest because of their extremely high contents and potential toxicity. The results obtained for these elements for 15, 30, 45, and 60 m distances from the points of contamination at two different depths (0–15 and 15–30 cm) are presented in Figure 2a,b for sampling sites #1 and #2, respectively. In view of the findings, it can be concluded that the whole studied area was characterized by relatively uniform pollution. Moreover, the analysis results (not shown) of soil from two presumably uncontaminated sites in the vicinity of the sampling areas still showed high concentrations of the studied elements.

In general, neither significant differences nor a particular trend in the concentration levels for any of the studied elements for the soils from sampling site #1 (Figure 2a) was observed, except for Ag, whose concentrations in the upper soil layer (0–15 cm) were regularly higher than in the lower (15–30 cm) one. In one particular sample (the upper layer of the soil taken at 60 m from the leach pad, the presumed contamination source), significantly higher (twice as high as the next highest value) concentrations of Pb, Ag, and Cd suggested the presence of a grain of rock enriched in these three elements. Among the elements studied, the most abundant metal was Pb, found at the average level of 2050 mg kg^−1^. Zinc concentration was in the range between 287 and 724 mg kg^−1^, and was at the same level as that observed for Cu concentration in the range between 245 and 512 mg kg^−1^, and As concentration was in the range between 252 and 401 mg kg^−1^. Much lower was the concentration of Ag, in the range between 4.1 and 20.7 mg kg^−1^, and of Cd, with an average concentration of 1.5 mg kg^−1^.

Concerning the results obtained for sampling site #2 (Figure 2b), a point with unusually high contents of Pb, Ag, and Cd was recorded in the upper layer of the mine dump (the presumed contamination source). Apart from this observation, no other clear trend in the measured concentrations was observed. In addition, for this sampling area (#2), lead was the metal yielding the highest concentrations with values between 934 and 2367 mg kg^−1^. The levels of Zn, Cu, and As remained comparable with the concentrations of Zn ranging from 120 to 305 mg kg^−1^, Cu from 131 to 283 mg kg^−1^, and As from 119 to 343 mg kg^−1^. Nevertheless, two exceptionally high values of ca. 730 mg kg^−1^ were recorded for As for both layers of the soil sample located farthest from the point of contamination. The average values for these three elements were significantly lower than in the case of sampling area #1. The total Ag concentration was in a range of several mg kg^−1^, and that of Cd was in general below 1 mg kg^−1^.

The Peruvian Environmental Quality Standards (PEQS) for soil set the maximum concentration permitted for Pb, As, and Cd in dried soils for agricultural uses at 70, 50, and 1.4 mg kg^−1^, respectively [44], which coincides with the Pb and Cd values described in the Canadian Soil Quality Guidelines (CSQG), being stricter for As with 12 mg kg^−1^ [45]. In sampling sites #1 and #2, lead was the metal with the highest concentrations considerably exceeding the maximum values allowed by soil regulations; likewise, As content also showed great excess. However, most Cd concentrations recorded low values, except in site #1 at the furthest distances from the point of contamination (45 and 60 m), where Cd values slightly exceeded the permitted standards. The PEQS do not contemplate maximum values for Zn, Cu, and Ag, unlike the Canadian soil standards, which set permitted values at 250, 63 and 20 mg kg^−1^, respectively [45,46]. According to Canadian standards, Zn exceeded the maximum allowed values in sampling site #1, whereas at sampling site #2 the concentration of Zn was above the legal threshold only at 0, 30, and 45 m away in the upper soil layer (0–15 cm). Copper concentrations significantly exceeded the maximum values allowed in both studied sites, and Ag was above the Canadian legal threshold only in samples from site #1 located furthest from the point of contamination (45 to 60 m). On the other hand, the Colquirrumi mine reported high content of heavy metals largely exceeding maximum allowed concentrations according to both PEQS and CSQG (Pb = 2069 mg kg^−1^, Zn = 1893 mg kg^−1^, Cu = 198 mg kg^−1^, As = 428 mg kg^−1^, and Cd = 13 mg kg^−1^) [22,39]. In addition, the Carolina mine, when it was an active mine in the same Hualgayoc district, reported high concentrations of Pb, Zn, Cu, and As (3992–16,060, 11,550–28,059, 256–2070, and 280–1030 mg kg^−1^, respectively) [47]. In addition, Table 2 shows concentration levels of Pb, Zn, Cu, As, Ag, and Cd found reported recently in other abandoned mines in Latin America, the majority of which are with acid soils.

Lead, As, Ag, Cd, as well as excess of Zn and Cu are toxic, nonbiodegradable and persist for long periods in the environment [6,21]. In strongly acidic conditions, as observed in our study case, these metals are released and cause potential hazards constituting a serious source of pollution to the surrounding areas [52], affecting directly or indirectly soil, water, flora, fauna, and human health [21]. The concentrations of Pb, Zn, As, Cu, Ag, and Cd studied in the mining environmental liabilities exceeded Peruvian and Canadian soil standards, suggesting a potential risk of the contamination. Thus, there is a vital need to implement sustainable techniques for the remediation of these mining sites [21].

### 3.3. Index of Geoaccumulation

**Figure 3:**
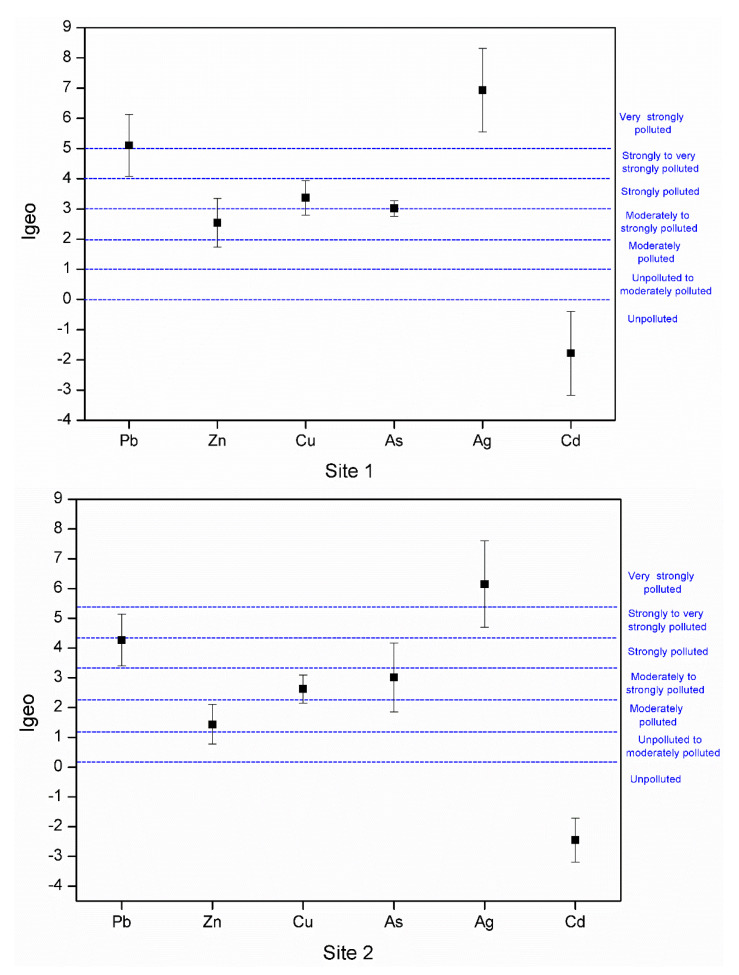
Geo-accumulation index (*I_geo_*) for Pb, Zn, Cu, As, Ag, and Cd at the sampling sites.

The degree of soil pollution in the studied areas was assessed using the index of geo-accumulation (*I_geo_*). *I_geo_* values for the studied metals are summarized in Figure 3. In general, the degree of pollution was similar in the two areas with results ranging from uncontaminated to extremely contaminated, depending on the metal. Considering Cd, the soils could be considered as unpolluted (*I_geo_* ≤ 0), whereas pollution with Zn was in the range from moderate to strong. Similarly, although higher pollution levels were observed for As and Cu. The highest *I_geo_* values were obtained for Pb (from strongly to very strongly contaminated) and for Ag (very strongly polluted). Finally, after the statistical analysis (detailed in Section 2.7) the calculated geo-accumulation index showed the following order of contamination levels for both examined areas: Cd < Zn < As ≅ Cu < Pb < Ag.

### 3.4. Sequential Extraction Study from the Soil

The total content of metals in soils does not fully reflect their potential environmental risk; from the ecological point of view, it is important to know which part of the total concentration of a metal is available to determine its toxic effect [33,34]. The proportions of metal fractions that determine the availability and mobility of trace elements in the soil and within the ecosystem, vary depending on the mineralogical composition of soils and other factors such as pH, organic matter content, and charge characteristics [53].

**Figure 4:**
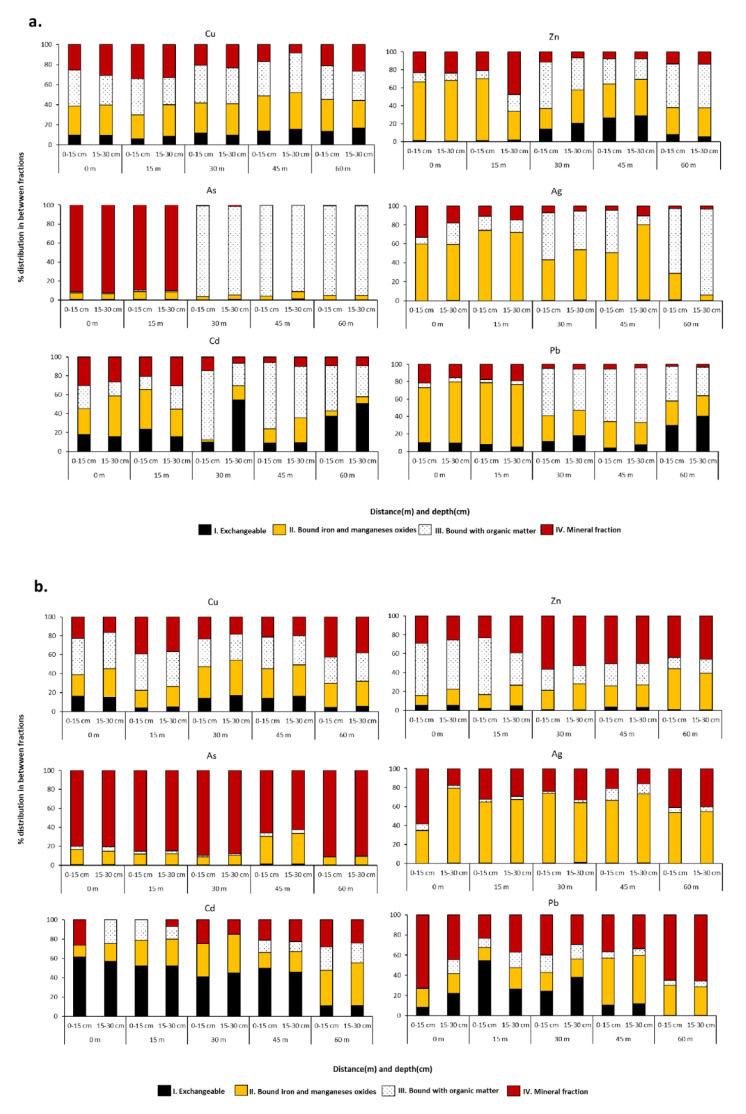
Percentage distribution of elements in four fractions by sequential extraction for five distances (0–60 m) and two depths (0–15 et 15–30 cm) from sampling area #1 (**a**) and #2 (**b**) in Hualgayoc district.

In our study, Pb, Zn, Cu, As, Ag, and Cd present in soil samples were separated into four fractions, providing a closer insight into their potential bioavailability. The fractions included (*i*) exchangeable metals forms, followed by metals bound to (*ii*) hydrated iron and manganese oxides, (*iii*) organic matter and, finally, (*iv*) mineral metal fraction. The partitioning of metals from the sampling areas #1 and #2 are presented in Figure 4a,b, respectively.

In general, no significant differences were found in the fractionation of Pb, Zn, Cu, As, Ag, and Cd between the upper (0–15 cm) and lower (15–30 cm) layers of soil. Out of 60 paired samples, only 6 (site #1: Zn—15 m, Ag—45 m and Cd—30 m and in site #2: Ag—0 m, Cd—0 m, Pb—0 m) displayed differences higher than +10% in the contributions of individual fractions into the total element content. The study of the distribution of elements in the soil showed the dominant presence of the fractions with limited metal mobility.

In sampling site #1, except for Cu, samples collected at 0 and 15 m from the leach pad presented notable differences from the three remaining ones collected at 30, 45, and 60 m. Copper was almost uniformly distributed among the mineral fraction and bound to organic matter and hydrated iron and manganese oxides; the remaining several percent of this metal was an exchangeable fraction. The two locations close to the presumed contamination source were characterized by the highest fraction of hydrated iron and manganese oxides-bound Pb, Ag, and Zn, whereas As was almost exclusively contained in the mineral fraction. Samples taken at 30, 45, and 60 m from the leach pad were characterized by a significant proportion of Pb, Ag, Zn, and As bound to organic matter, for which As reached more than 90% of its total content. The distribution of Cd was very variable with its total content distributed among all four fractions. The exchangeable fraction was absent for Ag, As, and Zn at 0 and 15 m from the leach pad. 

The fractionation of elements from sampling site #2 is shown in Figure 4b. In general, the distribution of elements among the fractions did not vary with the distance from the contamination source. Cadmium was the only of the studied metals for which the exchangeable fraction was present at significant proportions (between 20 and 60%). Most of As (70–90%) was contained in the mineral fraction, whereas the majority of Ag was bound to hydrated iron and manganese oxides. Zinc was distributed among three main fractions including mineral, organic matter, and hydrated iron and manganese oxides, similar as in the case of copper where, additionally, 5–20% of the exchangeable fraction was present. In this area, the distribution of Pb was complex and completely different than in area #1, with a significant portion represented by mineral forms.

The first exchangeable metal fraction is considered to contain the most mobile metal species and usually those that bestow surge to toxicity problems, being available as ions [53]. Indeed, our study indicates low exchange capacity of the studied soils. The potentially high bioavailability can be also expected for metal forms contained in organic matter [54]. Metal bound with oxides Fe-Mn represents one of the dominant fractions; it indicates the role of Fe or Mn oxides in the immobilization of metals similar to that in the residual fraction, which implies the binding of metals in minerals [55]. According to the available literature on contaminated soils, the phase distribution of trace metals is the highest in Fe-Mn oxide or in the residual fractions [40], from which metals are mostly unavailable to plants [54]. Despite the highest metals content being found in the fractions with limited mobility, the acidic conditions of the soils could favor their mobility and availability [53].

### 3.5. Trace Elements in Native Plants

Metals transport and distribution in plant tissues are impacted by the level of metal available and by the plant species [21]. In order to evaluate whether the native plants that grow in the two mining environmental liabilities could be indicators of potential metal mobility, metal concentration was evaluated in individual plant organs (leaves, stem, and roots). The results of the trace elements determination in organs of plants studied are given in Tables S1 and S2. Different plants have different abilities in the absorption of minerals, and the species that survive naturally in metalliferous soils are often only restricted to this type of area [56]. Metal availability can also be modified by plant roots through pH regulation affecting plant metal uptake [57,58]. Among the 21 plants collected, half of them were single specimens growing in one particular location, whereas for the other half, two to five individual specimens were found at different locations.

Zinc: The highest foliar Zn concentrations were found in the leaves of *Baccharis alnifolia* (172 mg kg^−1^—S1), *Achyrocline alata* (267 mg kg^−1^—S1 and 198 mg kg^−1^—S2), *Chusquea scandens* (153 mg kg^−1^—S1), and *Nicotiana thyrsiflora* (635 mg kg^−1^—S1). These values exceeded the threshold concentrations of 150 mg kg^−1^ [40]. The rest of the species yielded Zn concentrations within the normal values for the aerial parts of plants. However, ten species, *Hypericum laricifolium* (S1 and S2), *Pernettya prostrata* (S1 and S2), *Ageratina glechonophylla* (S1), *Cortaderia bifida* (S1 and S2), *Calceolaria tetragona* (S2), *Gaultheria glomerate* (S2), *Festuca* sp. (S2), *Calamagrostis recta* (S1), and *Arenaria digyna* (S1) had higher Zn concentrations in the roots. Plants growing in the Carolina mine area, geographically close to the two studied sites, accumulated higher quantities of Zn in their shoots (1424–16,192 mg kg^−1^) and roots (234–10,209 mg kg^−1^) irrespective of the species, of which, *Achyrocline alata*, one of the species in common with our study, accumulated in their shoots significant values (1423–6694 mg kg^−1^) [47]. This difference can be attributed to the different metal concentrations and the physicochemical characteristics of the soil, particularly pH, which was basic in soils studied by Bech et al. [47] in the Carolina mine, contrary to the highly acidic conditions in our case. In addition, other investigations in different shoot tissues reported 178–2008 mg kg^−1^ in Ecuador, 303–517 mg kg^−1^ in Chile, 11–144 mg kg^−1^ in Brazil, and 15–3414 mg kg^−1^ in Spain [47,56,59]. In our research between the two studied sites, four of the collected plant species—*Baccharis alnifolia, Achyrocline alata, Chusquea scandens*, and *Nicotiana thyrsiflora*—presented significant metal concentrations in their tissues, showing the ability to tolerate and accumulate toxic levels of Zn.

Copper: The concentrations of Cu in the majority of the native plants were the highest in the roots, of which, *Ageratina glechonophylla* (40 mg kg^−1^—S1), *Calamagrostis recta* (55 mg kg^−1^—S1), and *Arenaria digyna* (72 mg kg^−1^—S1) presented the most elevated values. On the other hand, species having a higher concentration of metal in the leaves than in the roots were *Achyrocline alata* (16 mg kg^−1^—S1 and 10 mg kg^−1^—S2) and *Hypericum laricifolium* (11 mg kg^−1^—S2). The only plant where the concentration in the aerial parts was higher than the normal range of 5–30 mg kg^−1^ [40] was *Nicotiana thyrsiflora* (49 mg kg^−1^—S1). Another study carried out close to our sampling area, at the Carolina mine, also reported high values in different plant species studied (38–542 mg kg^−1^ in shoots and 43–396 mg kg^−1^ in roots); moreover, the values reported for the shoots of *Achyrocline alata* (38–130 mg kg^−1^) [47] were higher, exceeding the range for aerial parts (30 mg kg^−1^). Studies in countries bordering Peru, such as Ecuador, Chile, and Brazil, showed Cu concentrations in shoots at 1–85, 77–988, and 32–137 mg kg^−1^, respectively [47,56]; likewise, other concentrations found were 40–243 mg kg^−1^ in Armenia (elevated) [38], and 20–29 mg kg^−1^ [60] in China. The results showed low Cu concentration in leaves, which may be due to low Cu translocation from roots to aerial parts, where it could interfere with photosynthesis and other essential processes [47].

Lead: In the present study, all species showed the highest Pb concentrations in the roots and the maximum value was found in *Arenaria digyna* (1658 mg kg^−1^–S1). In this species, Pb concentration in leaves (288 mg kg^−1^) exceeded the threshold value of 10 mg kg^−1^ for aerial parts [40]. Likewise, the concentrations in leaves of *Cortaderia bifida* (S1 and S2), *Calceolaria tetragona* (S2), *Ageratina fastigiate* (S1), *Hypericum laricifolium* (S1), *Achyrocline alata* (S1), *Puya* sp. (S1), *Orthrosanthus chimboracensis* (S1), *Nicotiana thyrsiflora* (S1), and *Ageratina glechonophylla* (S1) also exceeded the threshold value for aerial parts. Regardless of the plant species found in the Carolina mine, Pb concentrations were higher than in our study both in the roots (48–4841 mg kg^−1^) and in the shoots (346–6886 mg kg^−1^) [47]; also, the species common in both works, *Achyrocline alata*, showed higher concentration in shoots (1352–1650 mg kg^−1^) than roots (1319–1138 mg kg^−1^), presenting a different behavior than that found in our research, where most of the plants concentrate Pb in the roots and only some of them translocate it to aerial parts [61]. Previously, other studies reported the concentrations of Pb in shoots at the level of 11–614, 235–266, 0.12–341, and 7–9328 mg kg^−1^, respectively, in Ecuador, Chile, Spain, and Turkey [6,47,59].

Cadmium: In our study, Cd concentration in soil and also in the plants was relatively low, exceeding, however, the threshold value in aerial parts of 0.2 mg kg^−1^ [40]. In this group, some plants showed higher concentrations in leaves than in the roots: *Chusquea scandens* (0.39 mg kg^−1^—S1), *Orthrosanthus chimboracensis* (0.76 mg kg^−1^—S1), *Ageratina fastigiate* (1.40 mg kg^−1^—S1), *Baccharis alnifolia* (1.70 mg kg^−1^—S1), *Hypericum laricifolium* (1.88 mg kg^−1^—S1 and 2.53 mg kg^−1^—S2), *Achyrocline alata* (3.04 mg kg^−1^—S1), and *Nicotiana thyrsiflora* (21.62 mg kg^−1^—S1), the latter presenting an exceptionally high value. In addition, *Festuca* sp. (S2), *Gaultheria glomerate* (S2), *Calamagrostis recta* (S1), *Calceolaria tetragona* (S2), *Achyrocline alata* (S2), and *Arenaria digyna* (S1) also exceeded the normal concentration of Cd in aerial parts despite having the majority of Cd in their roots. Studies in Ecuador reported Cd content between 3–52 mg kg^−1^ in native plants [47]. Efficient inadvertent root absorption of Cd from contaminated soil was reported at mining sites in southern Morocco [21].

Arsenic: Arsenic levels in the majority of the studied species were higher in roots than in leaves. Three plants—*Puya* sp. (S1), *Calamagrostis recta* (S1), and *Arenaria digyna* (S1)—showed the highest concentrations (27, 42, and 53 mg kg^−1^, respectively). Several species, despite having a higher concentration of As in the roots, presented significant values in leaves, such as: *Hypericum laricifolium* (2.01 mg kg^−1^—S1), *Achyrocline alata* (3.82 mg kg^−1^—S1), *Arenaria digyna* (3.93 mg kg^−1^—S1), *Calamagrostis recta* (3.99 mg kg^−1^—S1), *Puya* sp. (5.12 mg kg^−1^—S1), *Ageratina glechonophylla* (6.44 mg kg^−1^—S1), and *Chusquea scandens* (4.76 mg kg^−1^—S1), exceeding the normal range of As (1.7 mg kg^−1^) for the aerial parts [40]. Low concentrations of As in shoots and roots in these native species differed from that reported in the Carolina mine, which were 21.3–298 mg kg^−1^ for shoots and 26–246 mg kg^−1^ for roots [47]. Also, *Achyrocline alata* (a species common with this study) had far higher values of 79–126 mg kg^−1^ in shoots and 64–74 mg kg^−1^ in roots, showing more As content in shoots than in roots. Other studies also reported high values in shoots: 22–343 mg kg^−1^ in Ecuador, 0.1–40 mg kg^−1^ in Spain, and 42–15,942 mg kg^−1^ in Turkey [6,47,59]. Lower concentrations of As in the native species observed in our study are most probably related to the virtual absence of the soil fractions allowing for the higher bioavailability, i.e., the exchangeable and carbonates-bound fractions [55].

Silver: Silver concentrations in all the studied native flora species were low, with roots showing higher contents than aerial parts. Nonetheless, some species exceeded the normal threshold of 0.5 mg kg^−1^ in their leaves [40], including *Ageratina glechonophylla* (0.75 mg kg^−1^—S1), *Brachyotum radula* (1.11 mg kg^−1^—S1), *Arenaria digyna* (2.28 mg kg^−1^—S1), and *Nicotiana thyrsiflora* (2.97 mg kg^−1^—S1). Previous studies showed Ag contents between 0.22–80 mg kg^−1^ [6]. The amount of Ag absorbed by plants is related to the concentration of soils; in many cases, Ag can be concentrated by plants to attain toxic levels [40]. Our study reported low Ag concentration in soil, and low percentage distribution of soil in the exchangeable and carbonate-bound fractions, thus the low concentration in the studied plants.

Trace elements concentrations in the investigated plants were very variable. In order to resist the toxic effects of metals, many plants developed a specific tolerance mechanism, such as restriction of metal translocation from roots into shoots [21]. High concentrations of As, Cd, Cu, Zn, and Pb were observed in shoots of plants from two botanical families: Poaceae (*Cortaderia Hapalotricha* and *Cortaderia nitida*), and Asteraceae (*Ageratina sp*, *Baccharis latifolia*, *Baccharis rhomboidalis*, *Baccharis amdatensis*) in Peru (the Carolina and Turmalina mines), Ecuador, and Chile by Bech et al. [47] where, however, metal concentrations were higher than in this study. It is interesting to mention that some studied plants, such as, e.g., *Hypericum laricifolium*, are used in traditional Peruvian medicine [62].

Some of the plant species were found at different sampling locations and, if the differences in soil metal content are not very high, can be considered as biological (quasi-) replicates. Indeed, these plants presented values reasonably close to each other (The relative standard deviation (RSD) for the large majority lower than 70%). The results are given in the Supplementary Information Table S3.

### 3.6. Bioconcentration (BCF) and Translocation Factor (TF) of the Native Plants

**Figure 5:**
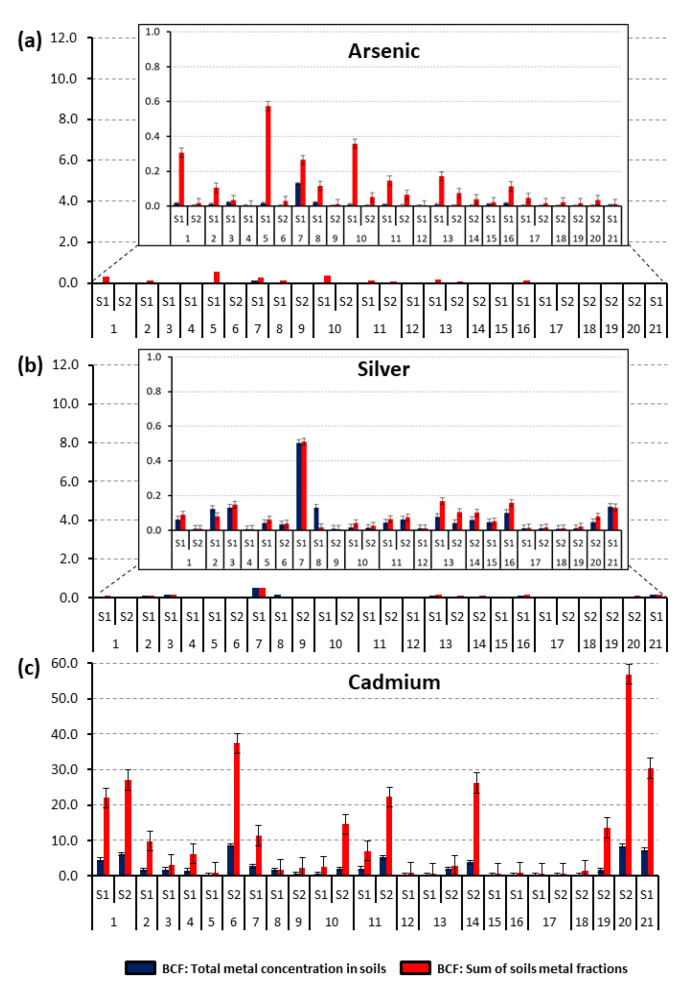

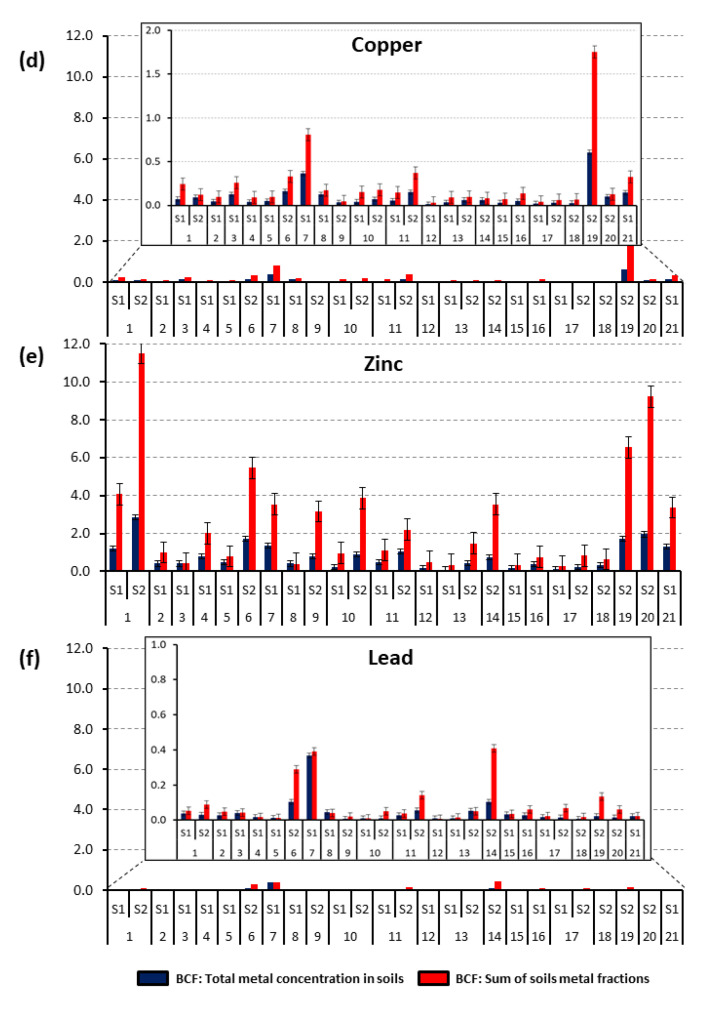
Bioconcentration factor (BCF) values of native species collected from two study areas (S1 and S2) in the Hualgayoc district. (**a**–**f**): 1—*Achyrocline alata*, 2—*Ageratina fastigiate*, 3—*Ageratina glechonophylla*, 4—*Baccharis alnifolia*, 5—*Puya sp*, 6—*Calceolaria tetragona*, 7—*Arenaria digyna*, 8—*Bejaria sp*, 9—*Gaultheria glomerata*, 10—*Pernettya prostrata*, 11—*Hypericum laricifolium*, 12—*Orthrosanthus chimboracensis,* 13—*Brachyotum radula*, 14—*Miconia vaccinioides*, 15—*Calamagrostis recta*, 16—*Chusquea scandens*, 17—*Cortaderia bifida*, 18—*Festuca sp*, 19—*Muehlenbeckia tamnifolia*, 20—*Buddleja interrupta*, 21—*Nicotiana thyrsiflora.*

**Figure 6:**
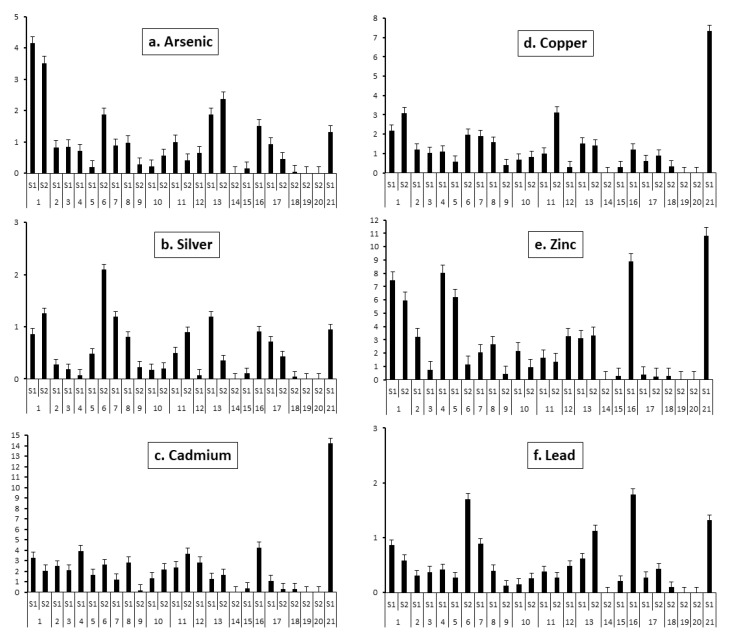
Translocation factor (TF) values of native species collected from two study areas (S1 and S2) in the Hualgayoc district. (**a**–**f**): 1—*Achyrocline alata*, 2—*Ageratina fastigiate*, 3—*Ageratina glechonophylla*, 4—*Baccharis alnifolia*, 5—*Puya sp*, 6—*Calceolaria tetragona*, 7—*Arenaria digyna*, 8—*Bejaria sp*, 9—*Gaultheria glomerata*, 10—*Pernettya prostrata*, 11—*Hypericum laricifolium*, 12—*Orthrosanthus chimboracensis,* 13—*Brachyotum radula*, 14—*Miconia vaccinioides*, 15—*Calamagrostis recta*, 16—*Chusquea scandens*, 17—*Cortaderia bifida*, 18—*Festuca sp*, 19—*Muehlenbeckia tamnifolia*, 20—*Buddleja interrupta*, 21—*Nicotiana thyrsiflora.*

Native plants of this study grow naturally around the mining environmental liabilities polluted with trace elements, demonstrating their good adaptation and tolerance to contaminated soil. The BCF and TF values reflected their metal accumulation and translocation. The BCF and TF values for the native Peruvian plants analyzed in this study are presented in Figure 5a–f and Figure 6a–f, respectively. The capacity of a plant to accumulate trace metals indicates its potential applicability for phytoextraction or phytostabilization process [38]. Phytoextraction requires the translocation of metals from soil to plant roots, whereas phytostabilization is the capacity to reduce metal translocation from roots to shoots [63]. A plant’s potential for phytoremediation can be estimated by the bioconcentration (BCF) and the translocation factor (TF) [37]; plants with these two indicators with values higher than one have potential to be used in phytoremediation. TF higher than one indicates a capacity to transport metal from roots to shoots, probably due to efficient metal transport systems and to the retention of metals in leaf vacuoles; low TF values show that more metals remain in the roots after plant uptake [20].

The BCF values were calculated with respect to the total metal concentration in soils (blue bars) and the sum of soils metal fractions with potential bioavailability (i.e., exchangeable and bound to carbonates, iron-manganese oxides, and organic matter; red bars). The part contained in the mineral fraction was considered as unavailable and, as such, not harmful from the point of view of environmental pollution. For As, Ag, Cu, and Pb for all the studied plants, the BCF values were <1 with the only exception of *Muehlenbeckia tamnifolia*, which was able to accumulate almost twice (1.76) the amount of bioavailable Cu. The factors influencing Cu uptake from contaminated soils and distribution in roots have been discussed in detail by Cui et al. [60]. Results of Cu translocation (Figure 5d) showed that in roughly half of the studied species, including *Hypericum laricifolium* (S1 and S2), *Ageratina glechonophylla* (S1), *Baccharis alnifolia* (S1), *Ageratina fastigiate* (S1), *Chusquea scandens* (S1), *Brachyotum radula* (S1 and S2), *Bejaria* sp. (S1), *Arenaria digyna* (S1), *Calceolaria tetragona* (S2), *Achyrocline alata* (S1 and S2), and *Nicotiana thyrsiflora* (S1), the TF index values were higher than one. No notable similarities in BCF values were observed for plants belonging to the same families: Asteraceae, Ericaceae, Hypericaceae, and Poaceae, for which several samples were available. This fact, however, can be due to the decisive role of individual variability of the plant specimens. Indeed, the role of the plant species seems to be important, which is demonstrated by significant variability in bioaccumulation capacities of plants growing in the same locations.

For Ag, TF values (Figure 6b) higher than one were obtained for *Brachyotum radula* (S1), *Arenaria digyna* (S1), *Calceolaria tetragona* (S2), and *Achyrocline alata* (S2). For the other plants, TF was lower than one. However, in all tested samples the BCF was <1, thus, no species was considered viable for Ag phytoremediation.

The translocation factor values for Pb were >1 (Figure 6f) in *Brachyotum radula* (S2), *Calceolaria tetragona* (S2), *Chusquea scandens* (S1), and *Nicotiana thyrsiflora* (S1), indicating that these four species can translocate metals from roots to the aerial part. These results are similar with previous studies, in which it was observed that the roots can absorb Pb from soils in a passive mode, where the rate of uptake is reduced by liming and low temperature, so that Pb translocation from roots to shoots is greatly limited [40] and, in consequence, Pb is not generally bioavailable in contaminated soil [63].

The BCF values for Zn were higher than one for the following plants species: *Muehlenbeckia tamnifolia* (S2), *Buddleja interrupta* (S2), *Nicotiana thyrsiflora* (S1), *Calceolaria tetragona* (S2), *Arenaria digyna* (S1), *Achyrocline alata* (S1 and S2), *Hypericum laricifolium* (S2). Zn TF > 1 was also observed for *Calceolaria tetragona* (S2), *Hypericum laricifolium* (S1 and S2), *Arenaria digyna* (S1), *Pernettya prostrata* (S1), *Bejaria* sp. (S1), *Brachyotum radula* (S1 and S2), *Ageratina fastigiate* (S1), *Orthrosanthus chimboracensis* (S1), *Achyrocline alata* (S1 and S2), *Puya* sp. (S1), *Baccharis alnifolia* (S1), *Chusquea scandens* (S1), and *Nicotiana thyrsiflora* (S1) (Figure 5e). It is noteworthy that plants such as *Baccharis alnifolia*, *Chusquea scandens*, and *Puya* sp. yielded very low BCF values despite having the highest TF, indicating that these species had difficulties in mobilizing this metal into roots [64].

The Cd BCF values were the highest out of all the metals studied; it has to be noted that according to the I_geo_ index, the studied area cannot be considered as contaminated by this element, so the enrichment led to relatively low concentrations in plant organs (in several plants, especially in the sampling area #2, the concentrations in aerial parts were lower than the threshold value of 0.2 mg kg^−1^ [40]). The majority of the species, including *Achyrocline alata* (S1 and S2), *Ageratina fastigiate* (S1), *Ageratina glechonophylla* (S1), *Baccharis alnifolia* (S1), *Arenaria digyna* (S1), *Calceolaria tetragona* (S2), *Bejaria* sp. (S1), *Hypericum laricifolium* (S1 and S2), *Brachyotum radula* (S2), *Muehlenbeckia tamnifolia* (S2), *Buddleja interrupta* (S2), *Nicotiana thyrsiflora* (S1), *Festuca* sp. (S2), *Pernettya prostrata* (S2), and *Miconia vaccinioides* (S2) presented BCF values higher than one. Cadmium TF value was higher than one for the majority (15 out of 21) of the species (Figure 6c): *Arenaria digyna* (S1), *Brachyotum radula* (S1 and S2), *Pernettya prostrata* (S1 and S2), *Puya* sp. (S1), *Achyrocline alata* (S1 and S2), *Ageratina glechonophylla* (S1), *Hypericum laricifolium* (S1 and S2), *Ageratina fastigiate* (S1), *Calceolaria tetragona* (S2), *Orthrosanthus chimboracensis* (S1), *Bejaria* sp. (S1), *Cortaderia bifida* (S1), *Baccharis alnifolia* (S1), *Chusquea scandens* (S1), and *Nicotiana thyrsiflora* (S1). Therefore, the species *Gaultheria glomerata* and *Festuca* sp., by having TF < 1 and BCF > 1, are considered good candidates for Cd phytostabilization. *Achyrocline alata* (S1 and S2), *Ageratina fastigiate* (S1), *Ageratina glechonophylla* (S1), *Baccharis alnifolia* (S1), *Arenaria digyna* (S1), *Calceolaria tetragona* (S2), *Bejaria* sp. (S1), *Pernettya prostrata* (S2), *Hypericum laricifolium* (S1 and S2), *Brachyotum radula* (S2), and *Nicotiana thyrsiflora* (S1), with TF >1 and BCF >1, can be considered adequate candidates for Cd phytoextraction.

For As, *Hypericum laricifolium* (S1), *Nicotiana thyrsiflora* (S1), *Chusquea scandens* (S1), *Calceolaria tetragona* (S2), *Brachyotum radula* (S1 and S2), and *Achyrocline alata* (S1 and S2), showed a TF index higher than one. However, for all the plant species, the BCF values were lower than one, so the studied plants can be considered as excluders [5], hence unsuitable for phytoremediation.

Among the plants studied, *Pernettya prostrata* and *Gaultheria glomerate* can be considered suitable for Zn phytostabilization, and *Achyrocline alata*, *Ageratina fastigiate*, *Baccharis alnifolia*, *Calceolaria tetragona*, *Arenaria digyna*, *Hypericum laricifolium*, *Brachyotum radula* and *Nicotiana thyrsiflora* for Zn phytoremediation. *Gaultheria glomerata* and *Festuca* sp. are also suitable for Cd phytostabilization, and *Achyrocline alata*, *Ageratina fastigiate*, *Ageratina glechonophylla*, *Baccharis alnifolia*, *Calceolaria tetragona*, *Arenaria digyna*, *Bejaria* sp., *Pernettya prostrata*, *Hypericum laricifolium*, *Brachyotum radula*, and *Nicotiana thyrsiflora* for Cd phytoremediation.

Bech et al. [47] reported in the Carolina mine at Hualgayoc (Cajamarca, Peru) that *Achyrocline alata* (common plant with this study) registered translocation factor values higher than one for Zn, Cu, Pb, and As metals; likewise, through shoot accumulation factor (SAF) this species was not considered as a good candidate for remediation purposes [43,47]. This research reported that for *A. alata*, TF index values were higher than one for Zn, Cu, Ag, Cd, and As, but not for Pb. Additionally, the BCF showed values <1 for Zn, Cu, Ag, As, and Pb, and >1 for Cd, so the authors considered *A. alata* as a good candidate for Cd phytoextraction. Additionally, in the Carolina mine plants from two botanical families such as Asteraceae (*Ageratina* sp. and *Baccharis latifolia*) and Poaceae (*Cortaderia Hapalotricha*) showed TF > 1 for As, Cu, Pb, and Zn, and were considered good candidates for remediation proposes for Pb and Zn [43,47,65]. However, although our study for the same botanical families—Asteraceae (*Ageratina fastigiate*, *Ageratina glechonophylla*, and *Baccharis alnifolia*) and Poaceae (*Cortaderia bifida*)—showed TF > 1 for Zn, Cu, and Cd (*A. fastigiate*), Cu and Cd (*A. glechonophylla*), Zn, Cu, and Cd (*B. alnifolia*), and Cd (*C. bifida*), none of the species had BCF > 1. Accordingly, these plants cannot be considered suitable for phytoremediation.

It has to be underlined, that the study of Bech et al. [47] concerned soils with basic pH, so despite the geographical proximity, the same plant species presented different behavior. In fact, the use of inorganic and organic amendments to acidic soil is recommended to improve metal removal efficiency by phytoremediation [66,67].

Plants with high TF and BCF values are able to extract metals from soils and transport them to the aerial parts. Plants with these characteristics could be considered as candidates for soil phytoremediation. If the BCF is high and the TF low, a plant can be used for phytostabilization [68]. Among the studied plants, *Festuca sp*, *Cortaderia bifida*, and *Gaultheria glomerate* can be considered suitable for Zn phytostabilization, and *Calceolaria tetragona* for Zn phytoremediation. *Gaultheria glomerata* and *Festuca* sp. are also suitable for Cd phytostabilization, and *Achyrocline alata*, *Arenaria digyna, Calceolaria tetragona*, and *Hypericum laricifolium* for Cd phytoremediation.

As for the BCF, no correlation could be found between the plants belonging to the same families: Asteraceae, Ericaceae, Hypericaceae, and Poaceae and their translocation capacity. However, for the same plant species, even growing at different locations (such as *Permettya prostrata*, *Hypericum laricifolium*, *Cortaderia bifida*, and *Brachyotum radula*), similar values were obtained. The plants showed similar behavior towards Pb and Ag and towards Cd, Cu, and Zn which, for the latter, is probably linked to the chemical proximity of these elements.

## 4. Conclusions

Native flora around two mining environmental liabilities from the Hualgayoc district in the Peruvian Andes was evaluated to assess their potential to accumulate metals in view of their possible use for the phytoremediation of post-mining sites. Soil samples collected at the two sampling sites showed the presence of high concentrations of Pb, Zn, Cu, As, Ag exceeding the levels of the Peruvian and Canadian regulations for agricultural soils, whereas the soils were not considered to be contaminated with Cd. On the other hand, the distribution of metals in the soil was studied, showing the dominant presence of fractions with limited metal mobility even if the acidic pH (observed in the studied area) is expected to increase the bioavailability of metals. 

The studied native plants appeared to be well-adapted to prosper in contaminated soils. Out of the 21 plants species evaluated, *Pernettya prostrata* and *Gaultheria glomerate* were suitable for Zn phytostabilization, and *Gaultheria glomerata* and *Festuca* sp. for Cd phytostabilization. It should be noted that the species *Gaultheria glomerata* is suitable for the phytostabilization of both Zn and Cd. *Cortaderia bifida* was the most abundant plant found at the two sampling areas. In addition, the native species applicable for Cd phytoremediation were *Achyrocline alata*, *Ageratina fastigiate*, *Ageratina glechonophylla*, *Baccharis alnifolia*, *Calceolaria tetragona*, *Arenaria digyna*, *Bejaria* sp., *Pernettya prostrata*, *Hypericum laricifolium*, *Brachyotum radula*, and *Nicotiana thyrsiflora*; and for that of Zn—*Achyrocline alata*, *Ageratina fastigiate*, *Baccharis alnifolia*, *Calceolaria tetragona*, *Arenaria digyna*, *Hypericum laricifolium*, *Brachyotum radula*, and *Nicotiana thyrsiflora*. It is noteworthy that all plant species found suitable for Zn phytoremediation could also be used for Cd phytoremediation. These plants have potential resistance to contaminated soils despite their limitations for growth and are, in consequence, a promising alternative for remediating contaminated soils in highlands.

## Figures and Tables

**Table 1:** Properties of soil samples from Hualgayoc district, Cajamarca region, Peru.

Distance (m)	Depth (cm)	pH	EC (dS m^−1^)	Carbonates (%)	Organic Matter (%)	Soil Texture (%)		
		R: <5.5–>8.8	R: <2–>8			Sand	Silt	Clay
**Sampling area #1**								
0	0–15	4.02	0.08	0	7.6	66	22	12
	15–30	3.97	0.09	0	4.3	50	24	26
15	0–15	4.19	0.05	0	4.4	52	26	22
	15–30	3.89	0.07	0	2.5	42	22	36
30	0–15	3.63	0.13	0	3.5	52	24	24
	15–30	3.84	0.15	0	3.8	54	28	18
45	0–15	3.63	0.08	0	5.7	66	18	16
	15–30	3.67	0.08	0	1.8	52	22	26
60	0–15	3.76	0.05	0	4.2	54	28	18
	15–30	3.59	0.09	0	4.5	50	24	26
**Sampling area #2**								
0	0–15	3.42	0.08	0	10.6	60	22	18
	15–30	3.37	0.11	0	5.0	56	24	20
15	0–15	4.02	0.02	0	4.4	54	20	26
	15–30	3.89	0.02	0	4.4	62	20	18
30	0–15	3.37	0.14	0	2.5	52	18	30
	15–30	3.46	0.14	0	2.9	54	18	28
45	0–15	3.85	0.05	0	4.9	56	22	22
	15–30	3.72	0.05	0	3.3	58	22	20
60	0–15	2.94	0.32	0	2.2	54	24	22
	15–30	2.77	0.57	0	1.7	54	20	26

EC: Electrical Conductivity/R: Range.

**Table 2:** Concentrations of Pb, Zn, Cu, As, Ag, and Cd (mg kg^−1^) reported in the literature for soil of mining sites in Peru and other South American countries.

Pb.	Zn	Cu	As	Ag	Cd	pH	pH Values	Name of the Mine	Status of the Mine	Country	Reference
3992–16,060	11,550–28,059	256–2070	280–1030	-	-	acid basic	6.8–8	Caroline mine-Hualgayoc	not active	Peru	[47]
87–341	56–772	69–5270	143–7670	-	8.9–499	acid	4.8	Turmaline mine-Piura	abandoned		
5.1–39.8	42–96	264- 977	-	-	-	acid	3.9	El teniente mine	active	Chile	
59–4890	58–18610	3–189	8.4–2240	1.6–178	2.9–342	acid	<4	San Bartolomé mine	abandoned	Ecuador	
-	-	1180–6310	-	-	-	acid	5.1–6.2	Petra verde mine	abandoned	Brazil	[48]
-	-	-	36–64	-	-	acid	4.5–5	Virgen Del Rosario and the Rayo Rojo cooperative mines	active artisanal	Bolivia	[49]
327–1754	448–505	149–459	183–14,660	-	45–308	acid basic	2.1–8.3	La Negra mine	abandoned	Mexico	[50]
780–43,700	380–>10,000	71.8–1320	19–11,800	9.5–74.2	1.0–780	acid	2.3–2.9	El Fraile mine	active	Mexico	[51]
670–4683	120–724	117–512	119–736	2.7–20.7	0.7–4.7	acid	2.8–4.9	Hualgayoc	abandoned	Peru	This work

## Supplementary Materials

The following are available online at https://www.mdpi.com/2223-7747/10/2/241/s1, Table S1. Chemical elements in the Earth’s Crust and geochemical baseline values near at the sampling sites. Table S2. Trace elements concentrations (mg kg^−1^) (mean ± SD) of As, Ag, Cd, Cu, Pb and Zn in different organs of the native plants from two study areas (S1 and S2) in the Hualgayoc district, Cajamarca region, Peru. Table S3. Average concentrations of trace elements in organs for the same plant species found in the same sampling area (S1 or S2).

## References

[1] Ozturk M., Altay V., Kucuk M., Ertuğrul Yalçın I. (2019). Trace Elements in the Soil-Plant Systems of Copper Mine Areas-A Case Study from Murgul Copper Mine from the Black Sea Region of Turkey. Phyton Int. J. Exp. Bot..

[2] Paredes M. (2016). The Glocalization of Mining Conflict: Cases from Peru. Extr. Ind. Soc..

[3] Figueroa B.E., Orihuela R.C., Calfucura T.E. (2010). Green Accounting and Sustainability of the Peruvian Metal Mining Sector. Resour. Policy.

[4] Ministerio de Energía y Minas (2018). Anuario Minero 2018.

[5] Lam E.J., Cánovas M., Gálvez M.E., Montofré Í.L., Keith B.F. (2017). Evaluation of the Phytoremediation Potential of Native Plants Growing on a Copper Mine Tailing in Northern Chile. J. Geochem. Explor..

[6] Yildirim D., Sasmaz A. (2017). Phytoremediation of As, Ag, and Pb in Contaminated Soils Using Terrestrial Plants Grown on Gumuskoy Mining Area (Kutahya Turkey). J. Geochem. Explor..

[7] Bouzekri S., El Hachimi M.L., Touach N., El Fadili H., El Mahi M., Lotfi E.M. (2019). The Study of Metal (As, Cd, Pb, Zn and Cu) Contamination in Superficial Stream Sediments around of Zaida Mine (High Moulouya-Morocco). J. Afr. Earth Sci..

[8] Aron A.S., Molina O. (2020). Green Innovation in Natural Resource Industries: The Case of Local Suppliers in the Peruvian Mining Industry. Extr. Ind. Soc..

[9] Manjate E., Ramos S., Almeida C.M.R. (2020). Potential Interferences of Microplastics in the Phytoremediation of Cd and Cu by the Salt Marsh Plant Phragmites Australis. J. Environ. Chem. Eng..

[10] Stefanowicz A.M., Kapusta P., Zubek S., Stanek M., Woch M.W. (2020). Soil Organic Matter Prevails over Heavy Metal Pollution and Vegetation as a Factor Shaping Soil Microbial Communities at Historical Zn-Pb Mining Sites. Chemosphere.

[11] Ministerio de Energía y Minas Law 28271 (2004). Ley Que Regula Los Pasivos Ambientales de La Actividad Minera. Diario Oficial El Peruano.

[12] Yupari A. (2003). Pasivos Ambientales Mineros En Sudamérica.

[13] Makhmudova G., Matsui K. (2019). The Remediation Policy after Mining Works in the Kyrgyz Republic. Resour. Policy.

[14] Ministerio de Energía y Minas Ministry Resolution N° 010-2019-MEM/DM (2019). Actualizan Inventario Inicial de Pasivos Ambientales Mineros. Diario Oficial El Peruano.

[15] Chappuis M. (2019). Remediación y Activación de Pasivos Ambientales Mineros (PAM) En El Perú. Medio Ambiente y Desarrollo N° 168.

[16] Pietrobelli C., Marin A., Olivari J. (2018). Innovation in Mining Value Chains: New Evidence from Latin America. Resour. Policy.

[17] Bian F., Zhong Z., Zhang X., Yang C., Gai X. (2020). Bamboo—An Untapped Plant Resource for the Phytoremediation of Heavy Metal Contaminated Soils. Chemosphere.

[18] Moreno-Jiménez E., Vázquez S., Carpena-Ruiz R.O., Esteban E., Peñalosa J.M. (2011). Using Mediterranean Shrubs for the Phytoremediation of a Soil Impacted by Pyritic Wastes in Southern Spain: A Field Experiment. J. Environ. Manag..

[19] Favas P.J.C., Pratas J., Varun M., Souza R.D., Paul M.S. (2014). Phytoremediation of Soils Contaminated with Metals and Metalloids at Mining Areas: Potential of Native Flora. Environmental Risk Assessment of Soil Contamination.

[20] Chaabani S., Abdelmalek-Babbou C., Ben Ahmed H., Chaabani A., Sebei A. (2017). Phytoremediation Assessment of Native Plants Growing on Pb-Zn Mine Site in Northern Tunisia. Environ. Earth Sci..

[21] Midhat L., Ouazzani N., Hejjaj A., Ouhammou A., Mandi L. (2019). Accumulation of Heavy Metals in Metallophytes from Three Mining Sites (Southern Centre Morocco) and Evaluation of Their Phytoremediation Potential. Ecotoxicol. Environ. Saf..

[22] Santos-Francés F., Martínez-Graña A., Rojo P.A., Sánchez A.G. (2017). Geochemical Background and Baseline Values Determination and Spatial Distribution of Heavy Metal Pollution in Soils of the Andes Mountain Range (Cajamarca-Huancavelica, Peru). Int. J. Environ. Res. Public Health.

[23] Canchaya S., Fontboté L., Amstutz C., Cardozo M., Cedillo E., Frutos J. (1990). Stratabound ore deposits of Hualgayoc, Cajamarca, Perú. Stratabound Ore Deposits in the Andes.

[24] Macfarlane A.W., Prol-Ledesma R.M., Conrad M.E. (1994). Isotope and Fluid Inclusion Studies of Geological and Hydrothermal Processes, Northern Peru. Int. Geol. Rev..

[25] Padilla W.G. (2019). Hualgayoc, Riqueza y Tradición.

[26] Macfarlane A.W., Petersen U. (1990). Pb Isotopes of the Hualgayoc Area, Northern Peru: Implications for Metal Provenance and Genesis of a Cordilleran Polymetallic Mining District. Econ. Geol..

[27] Bech J., Poschenrieder C., Barceló J., Lansac A. (2002). Plants from Mine Spoils in the South American Area as Potential Sources of Germplasm for Phytorenmediation Technologies. Acta Biotechnol..

[28] Walkley A., Black I.A. (1934). Estimation of Soil Organic Carbon by the Chromic Acid Titration Method. Soil Sci..

[29] Bouyoucos G.J. (1936). Directions for Making Mechanical Analyses of Soils by the Hydrometer Method. Soil Sci..

[30] Muller G. (1969). Index of Geo-Accumulation in Sediments of the Rhine River. Geojournal.

[31] Yaroshevsky A.A. (2006). Abundances of Chemical Elements in the Earth’s Crust. Geochem. Int..

[32] Okedeyi O.O., Dube S., Awofolu O.R., Nindi M.M. (2014). Assessing the Enrichment of Heavy Metals in Surface Soil and Plant (Digitaria Eriantha) around Coal-Fired Power Plants in South Africa. Environ. Sci. Pollut. Res..

[33] Tessier A., Campbell P.G.C., Bisson M. (1979). Sequential Extraction Procedure for the Speciation of Particulate Trace Metals. Anal. Chem..

[34] Głosińska G., Sobczyński T., Boszke L., Bierła K., Siepak J. (2005). Fractionation of Some Heavy Metals in Bottom Sediments from the Middle Odra River (Germany/Poland). Pol. J. Environ. Stud..

[35] Lago-Vila M., Arenas-Lago D., Rodríguez-Seijo A., Andrade M.L., Vega F.A. (2019). Ability of Cytisus Scoparius for Phytoremediation of Soils from a Pb/Zn Mine: Assessment of Metal Bioavailability and Bioaccumulation. J. Environ. Manag..

[36] Kamari A., Yusoff S.N.M., Putra W.P., Ishak C.F., Hashim N., Mohamed A., Phillip E. (2014). Metal Uptake in Water Spinach Grown on Contaminated Soil Amended with Chicken Manure and Coconut Tree Sawdust. Environ. Eng. Manag. J..

[37] Yoon J., Cao X., Zhou Q., Ma L.Q. (2006). Accumulation of Pb, Cu, and Zn in Native Plants Growing on a Contaminated Florida Site. Sci. Total Environ..

[38] Ghazaryan K., Movsesyan H., Ghazaryan N., Watts B.A. (2019). Copper Phytoremediation Potential of Wild Plant Species Growing in the Mine Polluted Areas of Armenia. Environ. Pollut..

[39] Cruzado-Tafur E., Torró L., Bierla K., Szpunar J., Tauler E. (2021). Heavy Metal Contents in Soils and Native Flora Inventory at Mining Environmental Liabilities in the Peruvian Andes. J. S. Am. Earth Sci..

[40] Kabata-Pendias A. (2011). Trace Elements in Soils and Plants.

[41] Tuo D., Xu M., Zhao Y., Gao L. (2015). Interactions between Wind and Water Erosion Change Sediment Yield and Particle Distribution under Simulated Conditions. J. Arid Land.

[42] Egerić M., Smičiklas I., Dojčinović B., Sikirić B., Jović M. (2019). Geoderma Interactions of Acidic Soil near Copper Mining and Smelting Complex and Waste-Derived Alkaline Additives. Geoderma.

[43] Bech J., Roca N., Tume P., Ramos-Miras J., Gil C., Boluda R. (2016). Screening for New Accumulator Plants in Potential Hazards Elements Polluted Soil Surrounding Peruvian Mine Tailings. Catena.

[44] Ministerio del Ambiente Supreme Decree N° 011-2017-MINAM (2017). Aprueban Estándares de Calidad Ambiental (ECA) Para Suelo. Diario Oficial El Peruano.

[45] Canadian Council of Ministers of the Environment (2007). Canadian Soil Quality Guidelines for the Protection of Environmental and Human Health: Summary Tables.

[46] Canadian Council of Ministers of the Environment (2018). Canadian Soil Quality Guidelines for the Protection of Environmental and Human Health: Zinc 2018.

[47] Bech J., Roca N., Tume P. (2017). Hazardous Element Accumulation in Soils and Native Plants in Areas Affected by Mining Activities in South America. Assessment, Restoration and Reclamation of Mining Influenced Soils.

[48] Perlatti F., Osório Ferreira T., Espíndola Romero R., Gomes Costa M.C., Otero X.L. (2015). Copper Accumulation and Changes in Soil Physical-Chemical Properties Promoted by Native Plants in an Abandoned Mine Site in Northeastern Brazil: Implications for Restoration of Mine Sites. Ecol. Eng..

[49] Acosta J.A., Arocena J.M., Faz A. (2015). Chemosphere Speciation of Arsenic in Bulk and Rhizosphere Soils from Artisanal Cooperative Mines in Bolivia. Chemosphere.

[50] Santos-Jallath J., Castro-Rodríguez A., Huezo-Casillas J., Torres-Bustillos L. (2012). Arsenic and Heavy Metals in Native Plants at Tailings Impoundments in Queretaro, Mexico. Phys. Chem. Earth.

[51] Herrera-Quiterio A., Toledo-Hernández E., Aguirre-Noyola J.L., Romero Y., Ramos J., Palemón-Alberto F., Toribio-Jiménez J. (2020). Antagonic and Plant Growth-Promoting Effects of Bacteria Isolated from Mine Tailings at El Fraile, Mexico. Revista Argentina de Microbiología.

[52] Yang S.X., Liao B., Yang Z.H., Chai L.Y., Li J.T. (2016). Revegetation of Extremely Acid Mine Soils Based on Aided Phytostabilization: A Case Study from Southern China. Sci. Total Environ..

[53] Arenas-Lago D., Andrade M.L., Lago-Vila M., Rodríguez-Seijo A., Vega F.A. (2014). Sequential Extraction of Heavy Metals in Soils from a Copper Mine: Distribution in Geochemical Fractions. Geoderma.

[54] He Z.L., Yang X.E., Stoffella P.J. (2005). Trace Elements in Agroecosystems and Impacts on the Environment. J. Trace Elem. Med. Biol..

[55] Ha N.T.H., Ha N.T., Nga T.T.H., Minh N.N., Anh B.T.K., Hang N.T.A., Duc N.A., Nhuan M.T., Kim K.W. (2019). Applied Geochemistry Uptake of Arsenic and Heavy Metals by Native Plants Growing near Nui Phao Multi-Metal Mine, Northern Vietnam. Appl. Geochem..

[56] França Afonso T., Faccio Demarco C., Pieniz S., Silveira Quadro M., Camargo F.A., Andreazza R. (2020). Bioprospection of Indigenous Flora Grown in Copper Mining Tailing Area for Phytoremediation of Metals. J. Environ. Manag..

[57] Claveria R.J.R., Perez T.R., Perez R.E.C., Algo J.L.C., Robles P.Q. (2019). The Identification of Indigenous Cu and As Metallophytes in the Lepanto Cu-Au Mine, Luzon, Philippines. Environ. Monit. Assess..

[58] Huang R., Dong M., Mao P., Zhuang P., Paz-Ferreiro J., Li Y., Li Y., Hu X., Netherway P., Li Z. (2020). Evaluation of Phytoremediation Potential of Five Cd (Hyper)Accumulators in Two Cd Contaminated Soils. Sci. Total Environ..

[59] Fernández S., Poschenrieder C., Marcenò C., Gallego J.R., Jiménez-Gámez D., Bueno A., Afif E. (2017). Phytoremediation Capability of Native Plant Species Living on Pb-Zn and Hg-As Mining Wastes in the Cantabrian Range, North of Spain. J. Geochem. Explor..

[60] Cui J., Zhao Y., Chan T., Zhang L., Tsang D.C.W., Li X. (2020). Spatial Distribution and Molecular Speciation of Copper in Indigenous Plants from Contaminated Mine Sites: Implication for Phytostabilization. J. Hazard. Mater..

[61] Hosseini S.M., Rezazadeh M., Salimi A., Ghorbanli M. (2018). Distribution of Heavy Metals and Arsenic in Soils and Indigenous Plants near an Iron Ore Mine in Northwest Iran. Acta Ecol. Sin..

[62] Vidal Ccana-Ccapatinta G., Lino von Poser G. (2015). Phytochemistry Letters Acylphloroglucinol Derivatives from Hypericum Laricifolium Juss. Phytochem. Lett..

[63] Drozdova I., Alekseeva-Popova N., Dorofeyev V., Bech J., Belyaeva A., Roca N. (2019). A Comparative Study of the Accumulation of Trace Elements in Brassicaceae Plant Species with Phytoremediation Potential. Appl. Geochem..

[64] Chandra R., Kumar V., Tripathi S., Sharma P. (2018). Heavy Metal Phytoextraction Potential of Native Weeds and Grasses from Endocrine-Disrupting Chemicals Rich Complex Distillery Sludge and Their Histological Observations during in-Situ Phytoremediation. Ecol. Eng..

[65] Bech J., Duran P., Roca N., Poma W., Sánchez I., Barceló J., Boluda R., Roca-Pérez L., Poschenrieder C. (2012). Shoot Accumulation of Several Trace Elements in Native Plant Species from Contaminated Soils in the Peruvian Andes. J. Geochem. Explor..

[66] Pedron F., Petruzzelli G., Barbafieri M., Tassi E. (2009). Strategies to Use Phytoextraction in Very Acidic Soil Contaminated by Heavy Metals. Chemosphere.

[67] Nagy A., Magyar T., Juhász C., Tamás J. (2020). Phytoremediation of Acid Mine Drainage Using By-Product of Lysine Fermentation. Water Sci. Technol..

[68] Xiao R., Shen F., Du J., Li R., Lahori A.H., Zhang Z. (2018). Screening of Native Plants from Wasteland Surrounding a Zn Smelter in Feng County China, for Phytoremediation. Ecotoxicol. Environ. Saf..

