# Supplementary material for: Accumulation of As, Ag, Cd, Cu, Pb, and Zn by Native Plants Growing in Soils Contaminated by Mining Environmental Liabilities in the Peruvian Andes
Source: Plants (Basel). 2021 Jan 27;10(2):241. doi: 10.3390/plants10020241 (PMC7922771; doi:10.3390/plants10020241)
Supplement: Supplementary file 1 [file plants-10-00241-s001.pdf]

## Supplementary Material

**Table S1.** Chemical elements abundances in the Earth's Crust and geochemical baseline values near at the sampling sites.

| | Pb | Zn | Cu | As | Ag | Cd |
|---|---|---|---|---|---|---|
| | (mg kg $^{-1}$) | | | | | |
| Abundances of Chemical Elements in the Earth's Crust [31] | 16 | 83 | 47 | 1.7 | 0.07 | 0.13 |
| Geochemical baseline values in a natural region (La Zanja) at 30km from the sampling place [22] | 44.87 | 47.42 | 22.2 | 27.5 | - | 4.36 |

**Table S2.** Trace elements concentrations (mg kg$^{-1}$) (mean ± SD) of As, As, Cd, Cu, Pb and Zn in different organs of the native plants from two study areas (S1 and S2) in Hualgayoc district, Cajamarca region, Peru.

| N° | Family | Native species | Sites | Biological replicates | Organs | Ag | As | Cd | Cu | Pb | Zn |
|---|---|---|---|---|---|---|---|---|---|---|---|
| 1 | Asteraceae | *Achyrocline alata* | S1 | 2 | L | 0.57 ± 0.02 | 3.82 ± 0.18 | 3.04 ± 0.35 | 15.6 ± 1.41 | 24.4 ± 2.69 | 266.9 ± 28.0 |
| | | | | | S | 0.15 ± 0.02 | 1.34 ± 0.05 | 3.58 ± 0.11 | 12.5 ± 0.25 | 38.2 ± 3.63 | 298.3 ± 26.3 |
| | | | | | R | 0.73 ± 0.09 | 1.75 ± 0.66 | 2.12 ± 0.50 | 12.5 ± 1.06 | 82.7 ± 6.11 | 76.6 ± 7.69 |
| | | | S2 | 1 | L | 0.06 ± 0.01 | 0.68 ± 0.03 | 2.03 ± 0.14 | 9.52 ± 0.74 | 8.65 ± 0.73 | 197.6 ± 8.45 |
| | | | | | S | 0.03 ± 0.01 | 0.20 ± 0.01 | 3.28 ± 0.12 | 11.4 ± 0.59 | 28.9 ± 3.67 | 181.5 ± 8.22 |
| | | | | | R | 0.08 ± 0.01 | 0.25 ± 0.01 | 2.59 ± 0.28 | 6.81 ± 0.15 | 64.1 ± 3.48 | 63.8 ± 2.57 |
| 2 | | *Ageratina fastigiate* | S1 | 3 | L | 0.50 ± 0.02 | 1.58 ± 0.12 | 1.40 ± 0.09 | 9.01 ± 1.35 | 13.8 ± 1.53 | 110.9 ± 27.6 |
| | | | | | S | 0.34 ± 0.04 | 1.85 ± 0.11 | 1.70 ± 0.16 | 7.12 ± 0.67 | 30.7 ± 4.09 | 71.6 ± 7.40 |
| | | | | | R | 3.09 ± 0.37 | 5.02 ± 0.72 | 1.26 ± 0.17 | 15.1 ± 1.63 | 139.1 ± 19.7 | 61.2 ± 5.47 |
| 3 | | *Ageratina glechonophylla* | S1 | 1 | L | 0.75 ± 0.04 | 6.44 ± 0.44 | 0.88 ± 0.01 | 29.5 ± 0.74 | 52.0 ± 3.93 | 80.4 ± 1.16 |
| | | | | | S | 0.12 ± 0.01 | 1.44 ± 0.14 | 1.81 ± 0.23 | 12.0 ± 0.24 | 25.1 ± 5.92 | 54.5 ± 1.57 |
| | | | | | R | 4.82 ± 0.21 | 9.25 ± 0.91 | 1.29 ± 0.13 | 40.3 ± 2.36 | 203.8 ± 13.3 | 177.4 ± 5.80 |
| 4 | | *Baccharis alnifolia* | S1 | 1 | L | 0.11 ± 0.01 | 0.38 ± 0.04 | 1.70 ± 0.12 | 7.12 ± 0.30 | 6.01 ± 0.82 | 171.6 ± 8.50 |
| | | | | | S | 0.06 ± 0.01 | 0.27 ± 0.01 | 3.45 ± 0.12 | 6.56 ± 0.60 | 66.4 ± 8.14 | 240.4 ± 29.0 |
| | | | | | R | 2.35 ± 0.17 | 0.90 ± 0.13 | 1.31 ± 0.09 | 12.3 ± 0.86 | 172.4 ± 22.3 | 51.3 ± 4.93 |
| 5 | Bromeliaceae | *Puya sp* | S1 | 1 | L | 0.48 ± 0.01 | 5.12 ± 0.91 | 0.22 ± 0.01 | 13.3 ± 0.82 | 28.5 ± 5.28 | 145.0 ± 2.57 |
| | | | | | R | 1.00 ± 0.08 | 26.84 ± 2.48 | 0.13 ± 0.02 | 22.6 ± 0.41 | 105.3 ± 11.8 | 23.38 ± 2.06 |
| 6 | Calceolariaceae | *Calceolaria tetragona* | S2 | 2 | L | 0.11 ± 0.01 | 0.52 ± 0.07 | 1.04 ± 0.13 | 7.22 ± 0.33 | 7.36 ± 0.64 | 53.08 ± 3.29 |
| | | | | | S | 0.10 ± 0.01 | 0.32 ± 0.02 | 6.63 ± 0.31 | 18.4 ± 0.87 | 104.0 ± 10.4 | 235.96 ± 10.2 |
| | | | | | R | 0.13 ± 0.01 | 0.72 ± 0.04 | 3.44 ± 0.06 | 12.7 ± 0.43 | 95.6 ± 5.06 | 220.65 ± 11.0 |

| N° | Family | Native species | Sites | Biological replicates | Organs | Ag | As | Cd | Cu | Pb | Zn |
|---|---|---|---|---|---|---|---|---|---|---|---|
| 7 | Caryophyllaceae | *Arenaria digyna* | S1 | 1 | L | 2.28 ± 0.10 | 3.93 ± 0.40 | 3.18 ± 0.20 | 54.3 ± 1.81 | 288.3 ± 32.6* | 342.34 ± 25.6 |
| | | | | | S | 10.49 ± 0.10 | 43.27 ± 6.49 | 6.30 ± 0.10 | 82.8 ± 1.65 | 1178.4 ± 46.5* | 458.15 ± 81.3* |
| | | | | | R | 10.69 ± 0.79 | 53.45 ± 3.49 | 7.71 ± 0.32 | 71.8 ± 3.31 | 1657.7 ± 187.2* | 413.38 ± 58.6* |
| 8 | Ericaceae | *Bejaria sp.* | S1 | 2 | L | 0.07 ± 0.01 | 0.62 ± 0.06 | 0.06 ± 0.01 | 4.37 ± 0.34 | 2.74 ± 0.47 | 17.63 ± 1.52 |
| | | | | | S | 0.03 ± 0.01 | 0.42 ± 0.06 | 0.35 ± 0.08 | 3.49 ± 0.55 | 9.42 ± 1.12 | 40.41 ± 10.8 |
| | | | | | R | 0.20 ± 0.05 | 1.10 ± 0.59 | 0.16 ± 0.03 | 3.70 ± 0.69 | 15.3 ± 6.62 | 25.02 ± 5.66 |
| 9 | | *Gaultheria glomerata* | S2 | 1 | L | 0.08 ± 0.01 | 0.52 ± 0.05 | 0.45 ± 0.02 | 7.61 ± 0.39 | 8.89 ± 1.02 | 104.0 ± 4.64 |
| | | | | | R | 0.35 ± 0.02 | 1.80 ± 0.19 | 2.75 ± 0.14 | 19.52 ± 0.47 | 72.62 ± 6.99 | 243.5 ± 12.0 |
| 10 | | *Pernettya prostrata* | S1 | 1 | L | 0.02 ± 0.01 | 0.68 ± 0.09 | 0.16 ± 0.01 | 4.64 ± 0.87 | 3.21 ± 1.09 | 28.8 ± 3.99 |
| | | | | | S | 0.12 ± 0.02 | 2.56 ± 0.54 | 0.54 ± 0.04 | 10.29 ± 1.32 | 16.9 ± 2.75 | 85.3 ± 14.79 |
| | | | | | R | 0.81 ± 0.14 | 15.19 ± 2.87 | 0.53 ± 0.06 | 22.01 ± 1.61 | 129.8 ± 20.71 | 52.8 ± 7.43 |
| | | | S2 | 2 | L | 0.03 ± 0.01 | 0.85 ± 0.02 | 0.42 ± 0.01 | 5.14 ± 0.38 | 3.34 ± 0.45 | 29.6 ± 0.73 |
| | | | | | S | 0.04 ± 0.01 | 0.55 ± 0.03 | 1.18 ± 0.03 | 7.50 ± 0.09 | 11.30 ± 1.46 | 117.57 ± 3.66 |
| | | | | | R | 0.36 ± 0.03 | 2.33 ± 0.14 | 0.78 ± 0.02 | 15.19 ± 0.41 | 55.45 ± 4.23 | 157.34 ± 6.43 |
| 11 | Hypericaceae | *Hypericum laricifolium* | S1 | 4 | L | 0.20 ± 0.03 | 2.01 ± 0.37 | 1.88 ± 0.04 | 9.71 ± 0.74 | 15.84 ± 2.06 | 101.9 ± 2.68 |
| | | | | | S | 0.11 ± 0.01 | 1.15 ± 0.20 | 1.25 ± 0.22 | 9.12 ± 0.95 | 35.42 ± 5.71 | 84.51 ± 6.76 |
| | | | | | R | 1.79 ± 0.23 | 4.40 ± 0.86 | 1.16 ± 0.17 | 21.53 ± 3.37 | 132.8 ± 18.61 | 133.6 ± 11.97 |
| | | | S2 | 1 | L | 0.12 ± 0.01 | 0.27 ± 0.01 | 2.53 ± 0.08 | 10.71 ± 0.24 | 3.77 ± 0.27 | 99.6 ± 8.06 |
| | | | | | S | 0.09 ± 0.01 | 0.37 ± 0.03 | 2.32 ± 0.06 | 8.21 ± 0.23 | 40.0 ± 2.29 | 127.5 ± 9.19 |
| | | | | | R | 0.23 ± 0.01 | 1.58 ± 0.17 | 1.33 ± 0.10 | 6.07 ± 0.68 | 159.6 ± 21.5 | 167.5 ± 11.38 |
| 12 | Iridaceae | *Orthrosanthus chimboracensis* | S1 | 1 | L | 0.25 ± 0.04 | 0.78 ± 0.04 | 0.76 ± 0.04 | 4.90 ± 0.23 | 33.3 ± 2.75 | 100.7 ± 6.56 |
| | | | | | R | 3.41 ± 0.22 | 1.21 ± 0.08 | 0.27 ± 0.03 | 15.92 ± 0.27 | 68.6 ± 9.00 | 30.9 ± 3.49 |
| 13 | Melastomalaceae | *Brachyotum radula* | S1 | 2 | L | 1.11 ± 0.04 | 1.71 ± 0.19 | 0.05 ± 0.01 | 6.83 ± 0.32 | 8.73 ± 0.62 | 32.4 ± 1.71 |
| | | | | | S | 0.58 ± 0.09 | 1.44 ± 0.43 | 0.12 ± 0.02 | 7.57 ± 0.34 | 8.97 ± 1.77 | 28.9 ± 1.58 |
| | | | | | R | 1.74 ± 0.21 | 3.87 ± 0.49 | 0.14 ± 0.01 | 11.4 ± 1.03 | 53.5 ± 5.72 | 19.2 ± 1.56 |
| | | | S2 | 2 | L | 0.17 ± 0.01 | 0.41 ± 0.03 | 0.07 ± 0.01 | 7.96 ± 0.12 | 2.60 ± 0.28 | 29.2 ± 1.44 |
| | | | | | S | 0.17 ± 0.01 | 0.53 ± 0.04 | 0.30 ± 0.03 | 9.60 ± 0.29 | 9.73 ± 1.04 | 41.7 ± 2.84 |
| | | | | | R | 1.18 ± 0.07 | 0.63 ± 0.11 | 0.39 ± 0.07 | 13.12 ± 0.29 | 16.9 ± 2.21 | 31.0 ± 1.28 |
| 14 | | *Miconia vaccinioides* [a] | S2 | 1 | L | 0.15 ± 0.01 | 0.26 ± 0.03 | 1.52 ± 0.06 | 10.65 ± 0.32 | 25.5 ± 1.66 | 70.4 ± 3.93 |
| | | | | | S | 0.09 ± 0.01 | 0.20 ± 0.02 | 1.58 ± 0.05 | 6.41 ± 0.23 | 76.6 ± 4.05 | 76.6 ± 2.62 |
| 15 | Poaceae | *Calamagrostis recta* | S1 | 2 | L | 0.67 ± 0.08 | 3.99 ± 0.40 | 0.55 ± 0.02 | 10.9 ± 0.50 | 100.4 ± 12.0 | 81.7 ± 5.16 |
| | | | | | R | 13.3 ± 0.76 | 42.4 ± 3.67 | 1.83 ± 0.13 | 54.7 ± 1.60 | 826.5 ± 120.6* | 309.5 ± 14.2 |
| 16 | | *Chusquea scandens* | S1 | 2 | L | 0.52 ± 0.07 | 4.76 ± 0.72 | 0.39 ± 0.01 | 11.5 ± 0.86 | 47.9 ± 5.44 | 152.5 ± 8.90 |
| | | | | | S | 0.43 ± 0.08 | 4.30 ± 0.87 | 0.19 ± 0.03 | 9.75 ± 2.13 | 36.1 ± 5.71 | 62.2 ± 8.81 |

| N° | Family | Native species | Sites | Biological replicates | Organs | Ag | As | Cd | Cu | Pb | Zn |
|---|---|---|---|---|---|---|---|---|---|---|---|
| | | | | | R | 1.97 ± 0.32 | 11.05 ± 2.24 | 0.25 ± 0.03 | 26.1 ± 2.35 | 77.6 ± 8.09 | 40.6 ± 0.98 |
| 17 | | *Cortaderia bifida* | S1 | 5 | L | 0.07 ± 0.01 | 0.49 ± 0.06 | 0.13 ± 0.10 | 4.15 ± 0.23 | 23.1 ± 2.73 | 25.5 ± 2.08 |
| | | | | | S | 0.08 ± 0.01 | 0.66 ± 0.04 | 0.17 ± 0.01 | 4.41 ± 0.41 | 22.3 ± 1.48 | 39.8 ± 8.27 |
| | | | | | R | 0.16 ± 0.04 | 1.35 ± 0.19 | 0.11 ± 0.01 | 7.96 ± 0.68 | 93.9 ± 10.4 | 99.3 ± 6.41 |
| | | | S2 | 5 | L | 0.05 ± 0.01 | 0.24 ± 0.01 | 0.07 ± 0.01 | 5.33 ± 0.20 | 17.0 ± 1.18 | 33.8 ± 1.17 |
| | | | | | S | 0.02 ± 0.01 | 0.17 ± 0.02 | 0.13 ± 0.01 | 3.45 ± 0.35 | 11.62 ± 0.54 | 23.7 ± 0.38 |
| | | | | | R | 0.19 ± 0.01 | 1.23 ± 0.08 | 0.30 ± 0.01 | 7.87 ± 0.29 | 48.43 ± 3.48 | 191.3 ± 13.3 |
| 18 | | *Festuca sp* | S2 | 1 | L | 0.03 ± 0.01 | 0.23 ± 0.01 | 0.31 ± 0.01 | 3.47 ± 0.07 | 5.18 ± 0.57 | 66.1 ± 1.20 |
| | | | | | R | 0.69 ± 0.03 | 4.33 ± 0.62 | 1.08 ± 0.06 | 10.36 ± 0.76 | 51.11 ± 7.85 | 251.1 ± 18.6 |
| 19 | Polygonaceae | *Muehlenbeckia tamnifolia* [a] | S2 | 1 | L | 0.05 ± 0.01 | 0.47 ± 0.01 | 1.05 ± 0.02 | 68.47 ± 1.86 | 17.10 ± 1.38 | 157.5 ± 3.38 |
| | | | | | S | 0.02 ± 0.01 | 0.26 ± 0.03 | 0.34 ± 0.02 | 21.33 ± 1.41 | 25.34 ± 3.31 | 65.6 ± 4.50 |
| 20 | Scrophulariaceae | *Buddleja interrupta* [a] | S2 | 1 | L | 0.14 ± 0.03 | 0.24 ± 0.04 | 2.37 ± 0.24 | 11.92 ± 0.36 | 1.42 ± 0.20 | 142.0 ± 8.49 |
| | | | | | S | 0.05 ± 0.01 | 0.15 ± 0.01 | 4.37 ± 0.13 | 15.38 ± 0.17 | 13.7 ± 1.31 | 241.1 ± 10.8 |
| 21 | Solanaceae | *Nicotiana thyrsiflora* | S1 | 1 | L | 2.97 ± 0.22 | 1.70 ± 0.03 | 21.62 ± 0.25 | 48.51 ± 0.57 | 41.8 ± 2.05 | 635.4 ± 128.9* |
| | | | | | S | 0.37 ± 0.03 | 0.29 ± 0.03 | 3.70 ± 0.29 | 6.30 ± 0.34 | 45.0 ± 5.29 | 245.0 ± 30.7* |
| | | | | | R | 3.52 ± 0.05 | 1.52 ± 0.09 | 1.78 ± 0.09 | 7.47 ± 0.09 | 66.1 ± 9.11 | 71.5 ± 6.66 |

L: leaf, S: Stem, R: Root. *Outlier, not taken into statistical processing. [a] Species collected without roots, not taken for the analysis of the results.

**Table S3.** Average concentrations of trace elements in organs for the same plant species found in the same sampling area (S1 or S2).

| Plant species | Sampling area | Nr of biological replicates | Organs | Ag mg/kg ± SD | As mg/kg ± SD | Cd mg/kg ± SD | Cu mg/kg ± SD | Pb mg/kg ± SD | Zn mg/kg ± SD |
|---|---|---|---|---|---|---|---|---|---|
| *Ageratina fastigiate* | 1 | 3 | L | 0,50 ± 0,36 | 1,58 ± 1,31 | 1,40 ± 0,18 | 9,0 ± 0,9 | 111,0 ± 32,7 | 13,8 ± 5,0 |
| | | | S | 1,85 ± 0,34 | 1,85 ± 1,69 | 1,70 ± 0,36 | 7,1 ± 2,1 | 71,6 ± 14,1 | 30,7 ± 24,5 |
| | | | R | 3,09 ± 2,69 | 5,02 ± 4,51 | 1,26 ± 0,22 | 15,1 ± 7,1 | 61,2 ± 25,3 | 139,1 ± 78,3 |
| *Cortaderia bifida* | 1 | 5 | L | 0,49 ± 0,29 | 0,07 ± 0,13 | 0,13 ± 0,17 | 4,1 ± 0,9 | 25,5 ± 0,8 | 23,1 ± 24,3 |
| | | | R | 1,35 ± 1,38 | 0,16 ± 0,11 | 0,11 ± 0,04 | 8,0 ± 2,7 | 99,3 ± 6,9 | 93,9 ± 20,2 |
| *Achyrocline alata* | 1 | 2 | L | 3,82 ± 1,78 | 0,57 ± 0,63 | 3,04 ± 2,49 | 15,6 ± 8,5 | 267,0 ± 193,9 | 24,4 ± 7,9 |
| | | | S | 1,34 ± 0,53 | 0,15 ± 0,06 | 3,58 ± 1,86 | 12,5 ± 4,0 | 298,4 ± 47,2 | 38,2 ± 6,7 |
| | | | R | 1,75 ± 1,19 | 0,73 ± 0,31 | 2,12 ± 1,62 | 12,5 ± 2,6 | 76,6 ± 26,0 | 82,7 ± 40,8 |
| *Hypericum laricifolium* | 1 | 4 | L | 2,01 ± 0,47 | 0,2 ± 0,05 | 1,88 ± 2,61 | 9,7 ± 2,0 | 101,8 ± 86,4 | 15,8 ± 5,8 |
| | | | S | 1,15 ± 0,80 | 0,11 ± 0,01 | 1,25 ± 1,47 | 9,1 ± 3,8 | 84,5 ± 65,4 | 35,4 ± 10,8 |
| | | | R | 4,40 ± 2,61 | 1,79 ± 0,33 | 1,16 ± 1,41 | 21,5 ± 10,5 | 133,6 ± 138,5 | 132,8 ± 11,4 |
| *Calamagrostis recta* | 1 | 2 | L | 3,99 ± 2,17 | 0,67 ± 0,41 | 0,55 ± 0,48 | 10,9 ± 0,2 | 81,7 ± 19,4 | 100,4 ± 87,8 |

| | | | | | | | | | |
|---|---|---|---|---|---|---|---|---|---|
| | | | R | 42,38 ± 45,75 | 13,3 ± 15,9 | 1,83 ± 1,89 | 54,7 ± 43,0 | 309,5 ± 38,5 | 826,5 ± 96,8 |
| | | | L | 1,71 ± 0,11 | 1,11 ± 1,09 | 0,05 ± 0,00 | 6,8 ± 0,8 | 32,4 ± 20,9 | 8,7 ± 1,7 |
| *Brachyotum radula* | 1 | 2 | S | 1,44 ± 1,27 | 0,58 ± 0,59 | 0,12 ± 0,01 | 7,6 ± 1,7 | 28,9 ± 13,5 | 9,0 ± 1,0 |
| | | | R | 3,87 ± 3,77 | 1,74 ± 0,56 | 0,14 ± 0,01 | 11,4 ± 6,8 | 19,2 ± 8,7 | 53,6 ± 51,2 |
| *Chusquea scandens* | 1 | 2 | L | 4,76 ± 5,55 | 0,52 ± 0,64 | 0,39 ± 0,48 | 11,5 ± 8,6 | 152,6 ± 163,3 | 47,9 ± 44,4 |
| | | | S | 4,30 ± 5,35 | 0,43 ± 0,53 | 0,19 ± 0,18 | 9,7 ± 6,4 | 62,3 ± 42,0 | 36,1 ± 33,3 |
| *Bejaria sp.* | 2 | 2 | L | 0,62 ± 0,25 | 0,07 ± 0,06 | 0,06 ± 0,08 | 4,4 ± 1,4 | 17,6 ± 1,3 | 2,7 ± 0,3 |
| | | | S | 0,42 ± 0,19 | 0,03 ± 0,03 | 0,35 ± 0,23 | 3,5 ± 1,5 | 40,4 ± 10,7 | 9,4 ± 8,8 |
| *Cortaderia bifida* | 2 | 5 | L | 2,45 ± 1,82 | 0,50 ± 0,43 | 0,46 ± 0,49 | 8,6 ± 3,0 | 73,8 ± 53,0 | 39,04 ± 39,0 |
| | | | R | 11,07 ± 1,44 | 3,47 ± 0,64 | 0,73 ± 0,23 | 20,2 ± 2,9 | 113,0 ± 30,6 | 211,68 ± 39,4 |
| | | | L | 0,85 ± 0,82 | 0,03 ± 0,003 | 0,42 ± 0,03 | 5,1 ± 2,3 | 29,6 ± 2,7 | 3,34 ± 1,25 |
| *Pernettya prostrata* | 2 | 2 | S | 0,55 ± 0,29 | 0,04 ± 0,001 | 1,18 ± 0,08 | 7,5 ± 2,2 | 117,6 ± 32,0 | 11,2 ± 2,73 |
| | | | R | 2,33 ± 1,21 | 0,36 ± 0,03 | 0,78 ± 0,27 | 15,2 ± 3,1 | 157,3 ± 40,1 | 55,4 ± 14,5 |
| | | | L | 0,52 ± 0,47 | 0,11 ± 0,1 | 1,04 ± 0,35 | 7,2 ± 1,8 | 53,1 ± 12,4 | 7,36 ± 8,2 |
| *Calceolaria tetragona* | 2 | 2 | S | 0,32 ± 0,25 | 0,10 ± 0,001 | 6,63 ± 2,14 | 18,4 ± 2,5 | 236,0 ± 30,8 | 104,0 ± 64,6 |
| | | | R | 0,72 ± 0,09 | 0,13 ± 0,03 | 3,44 ± 1,88 | 12,7 ± 3,0 | 220,6 ± 124,7 | 95,6 ± 58,2 |
| | | | L | 0,54 ± 0,02 | 0,08 ± 0,05 | 1,11 ± 0,10 | 7,4 ± 0,2 | 85,3 ± 45,6 | 9,33 ± 2,8 |
| *Brachyotum radula* | 2 | 2 | S | 1,32 ± 1,42 | 0,23 ± 0,18 | 3,71 ± 4,14 | 16,8 ± 2,3 | 196,7 ± 55,6 | 79,7 ± 34,3 |
| | | | R | 0,72 ± 0,14 | 0,13 ± 0,02 | 3,44 ± 1,70 | 12,7 ± 3,8 | 220,6 ± 118,5 | 95,6 ± 62,4 |

L: leaf, S: Stem, R: Root.
